# Deciphering the Synthetic and Refolding Strategy of a Cysteine-Rich Domain in the Tumor Necrosis Factor Receptor (TNF-R) for Racemic Crystallography Analysis and d-Peptide Ligand Discovery

**DOI:** 10.1021/acsbiomedchemau.3c00060

**Published:** 2023-12-11

**Authors:** Alexander J. Lander, Yifu Kong, Yi Jin, Chuanliu Wu, Louis Y. P. Luk

**Affiliations:** § School of Chemistry, Cardiff University, Main Building, Park Place, Cardiff CF10 3AT, U.K.; † Department of Chemistry, College of Chemistry and Chemical Engineering, The MOE Key Laboratory of Spectrochemical Analysis and Instrumentation, State Key Laboratory of Physical Chemistry of Solid Surfaces, Xiamen University, Fujian Province 361005, China; ‡ Manchester Institute of Biotechnology, University of Manchester, Manchester M1 7DN, U.K.

**Keywords:** Chemical protein synthesis, protein folding, cysteine-rich protein, mirror-image phage display, racemic protein crystallography, d-protein, d-peptide

## Abstract

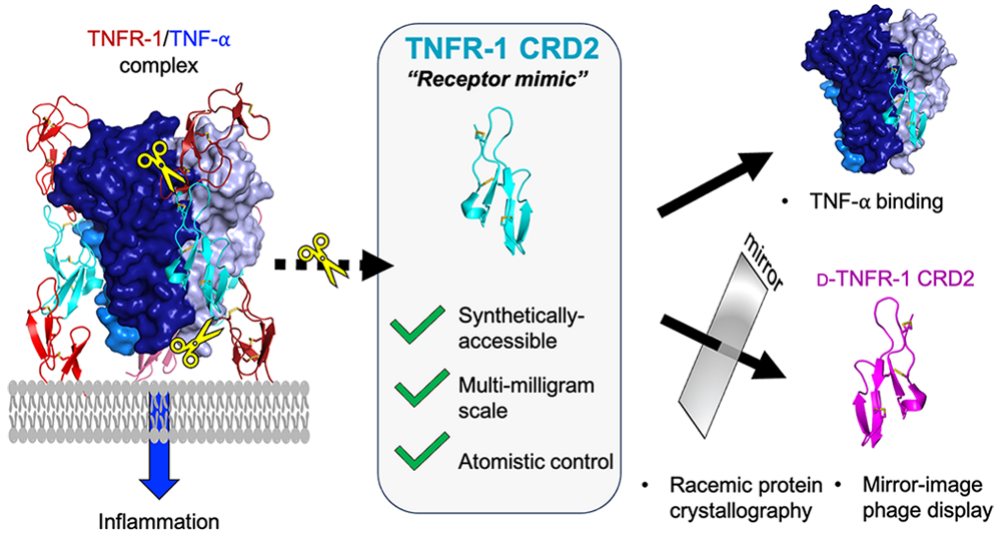

Many cell-surface receptors are promising targets for chemical synthesis because of their critical roles in disease development. This synthetic approach enables investigations by racemic protein crystallography and ligand discovery by mirror-image methodologies. However, due to their complex nature, the chemical synthesis of a receptor can be a significant challenge. Here, we describe the chemical synthesis and folding of a central, cysteine-rich domain of the cell-surface receptor tumor necrosis factor 1 which is integral to binding of the cytokine TNF-α, namely, TNFR-1 CRD2. Racemic protein crystallography at 1.4 Å confirmed that the native binding conformation was preserved, and TNFR-1 CRD2 maintained its capacity to bind to TNF-α (*K*_D_ ≈ 7 nM). Encouraged by this discovery, we carried out mirror-image phage display using the enantiomeric receptor mimic and identified a d-peptide ligand for TNFR-1 CRD2 (*K*_D_ = 1 μM). This work demonstrated that cysteine-rich domains, including the central domains, can be chemically synthesized and used as mimics for investigations.

## Introduction

Cell-surface receptors, responsible for converting external stimuli to cellular responses, are critical targets for physiological investigations as well as diagnostics and therapeutics design.^1^ While recombinant routes for preparing receptors are most frequently used, chemical protein synthesis has unique advantages for research by offering atomistic control and fully breaking the barrier of chirality.^2^ In particular, chemical synthesis enables preparation of enantiomeric proteins entirely composed of d-amino acids,^3^ and hence, racemic protein crystallography can be conducted for high-resolution structural analysis.^4^ Furthermore, mirror-image display methods can be performed for discovery of ultrastable d-peptide and l-RNA aptamer ligands.^5^ Despite these exciting possibilities, the chemical synthesis of cell-surface receptors presents a significant challenge.^6^ Many cell-surface receptors are lengthy, heavily post-translationally modified, and possess patches of hydrophobic segments that are prone to aggregate formation. This often results in low yields and potential contamination from misfolded byproducts.^6^

**Figure 1:**
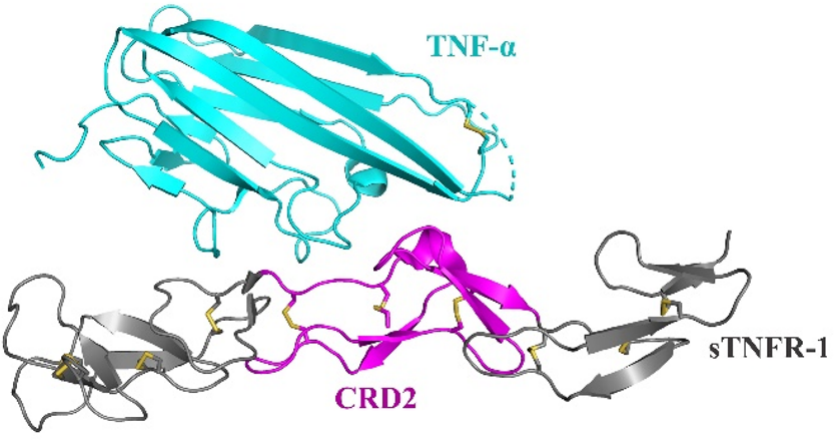
Structure of the TNFR-1/TNF-α complex (PDB: 7KPB) highlighting the cysteine-rich domain 2 (CRD2, magenta) prepared in this work.

In order to simplify the process of chemical receptor synthesis, it is possible to focus on preparing a smaller but functionally relevant domain of the protein, such as terminal protein fragments^7^ or short peptide mimics.^8^ Moreover, it is important to note that, while the ligand binding interfaces are arguably most crucial for investigations, they are often confined within the central portion of the extracellular receptors.^9^ One notable example is the tumor necrosis factor receptor I (TNFR-1, UniProt P19438), a key drug target in various types of inflammatory diseases.^1a,10^ Consisting of 144 residues and 12 disulfide bonds as post-translation modifications, the extracellular domain of TNFR-1 presents a challenging synthetic target. Nevertheless, the binding of TNFR-1 to its natural ligand TNF-α (UniProt P01375) primarily occurs at the central portion of the cysteine-rich domain 2 (CRD2) (Figure 1),^11^ which contains three disulfide bonds and two pairs of beta-sheets. As this central portion of the receptor contains a significant number of TNF-α interface residues,^11b^ we envisioned that it can be synthesized individually and serve as a truncated mimic for the TNFR-1.

Here, we present a synthetic route to the TNFR-1 CRD2 protein domain using a one-pot chemical ligation and refolding procedure. The resulting construct manifested a significant affinity for the native TNF-α cytokine (*K*_D_ ≈ 7 nM). The structure of TNFR-1 CRD2, determined through racemic protein crystallography, revealed the proper formation of the three disulfide bonds and showed good alignment of its backbone with the structure of the full receptor, exhibiting a backbone RMSD of 0.36 Å (PDB: 7KPB). Motivated by these observations, the enantiomeric TNFR-1 CRD2, entirely composed of d-amino acid building blocks, served as a receptor mimic in a mirror image phage display, yielding the identification of a cyclic d-peptide with a binding affinity (*K*_D_) of 1.0 μM for the receptor mimic with natural l-chirality.

## Results and Discussion

### Synthesis and Folding of TNFR-1 CRD2

**Figure 2:**
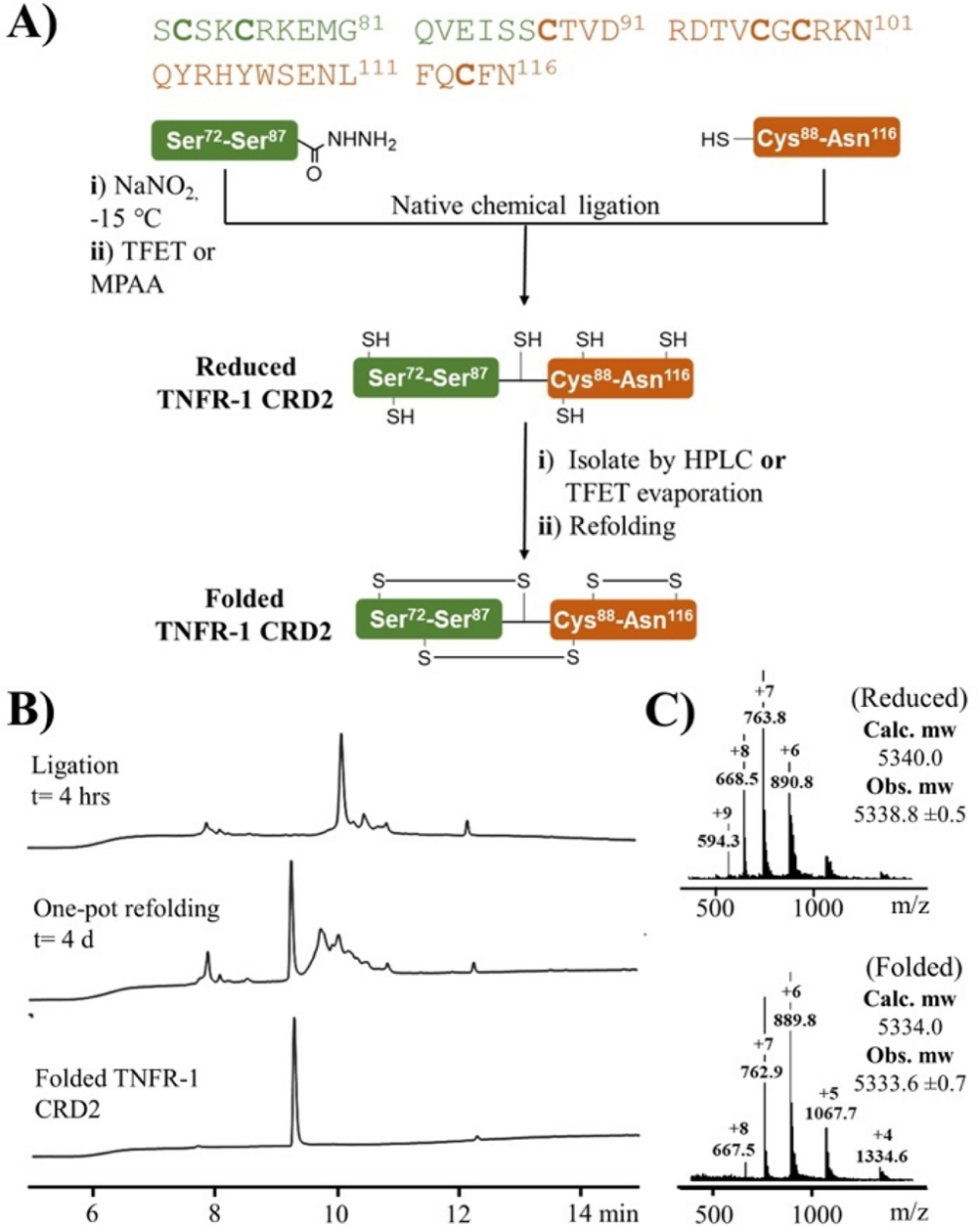
(A) Amino acid sequence and synthetic route to TNFR-1 CRD2, (B) HPLC traces monitoring one-pot ligation and refolding reaction of TNFR-1 CRD2, and (C) ESI+ mass spectra of reduced (top) and folded (bottom) TNFR-1 CRD2.

The preparation of the entire 45-residue TNFR-1 CRD2 polypeptide by a solid-phase peptide synthesis (SPPS) approach was first attempted (Figure 2A). However, large excesses of amino acid and repeated coupling reactions were needed at around the 30th residue (Ser72-Ser86; data not shown). This included the diastereomeric isoleucine (Ile85), which is notably costly in its d-enantiomeric form (Fmoc-d-Ile-OH £231/g, Fmoc-l-Ile-OH < £1.32/g, Sigma-Aldrich). Asp93 was also found to be prone to form aspartimide. Lastly, incorporation of the flexible C-terminal linker for immobilization (GSGSGK(biotin)) further hampered the yield in the “one-shot” approach. Accordingly, we developed a synthetic approach based on native chemical ligation (NCL; Figure 2A).^12^ In this strategy, the N-terminal peptide containing the first 16 residues is prepared as a peptide hydrazide, which is then oxidized and converted to a C-terminal thioester in situ.^13^ The remaining 29 residues were incorporated using the N-terminal cysteine peptide, either alone or with a C-terminal biotin linker. Subsequently, both peptides were amenable to SPPS using just 2 equiv of amino acids during couplings, and the strategic positioning of Asp93 toward the end of the peptide chain allowed minimization of aspartimide formation (see Supporting Information S6). The ligation reaction was initially catalyzed by 4-mercaptophenylacetic acid (MPAA)^14^ and the resulting product was isolated via preparative HPLC for further optimization of the refolding conditions.

As the linear TNFR-1 CRD2 polypeptide contains six cysteine residues, there are 15 possible combinations for disulfide bond formation. Refolding of synthetic cysteine-rich miniproteins is often case dependent, even when structural prediction programme such as AlphaFold 2 could accurately predict the structure of the folded protein.^7b,15^ Therefore, to identify the most thermodynamically stable conformer, various refolding conditions were screened, including different oxidative agents (air, DMSO, glutathione disulfide), buffer (sodium phosphate at pH 6.5, Tris at pH 8.5), temperature (4, 25 °C), and reagent concentrations (oxidative agents, protein) (Supporting Information S1). Each refolding trial involved the rapid dilution of the reduced protein. The reactions were monitored by LCMS, seeking for an upfield peak that contains a 6 Da mass loss of the oxidatively refolded miniprotein. The highest refolding yield (33% by HPLC) was achieved by preparing a solution of TNFR-1 CRD2 (0.5 mg/mL) in 6 M Gdn·HCl, 0.1 M sodium phosphate buffer (pH 6.5), 6 mM glutathione disulfide, and 60 mM glutathione, which was then rapidly diluted 5-fold with 0.1 M phosphate buffer (pH 6.5) (Table S1.1). To simplify the synthetic scheme, a one-pot approach combining ligation and refolding was developed (Figure 2B). In this approach, trifluoroethanthiol (TFET) was used as a thiol catalyst instead of MPAA.^16^ TFET, being volatile (boiling point 35–37 °C), could be easily removed by evaporation,^16^ allowing immediate refolding after ligation without purification and giving a yield of 17% (Figure 2B and C).

### Validation of the Isolated TNFR-1 CRD2 as a Receptor Mimic

**Figure 3:**
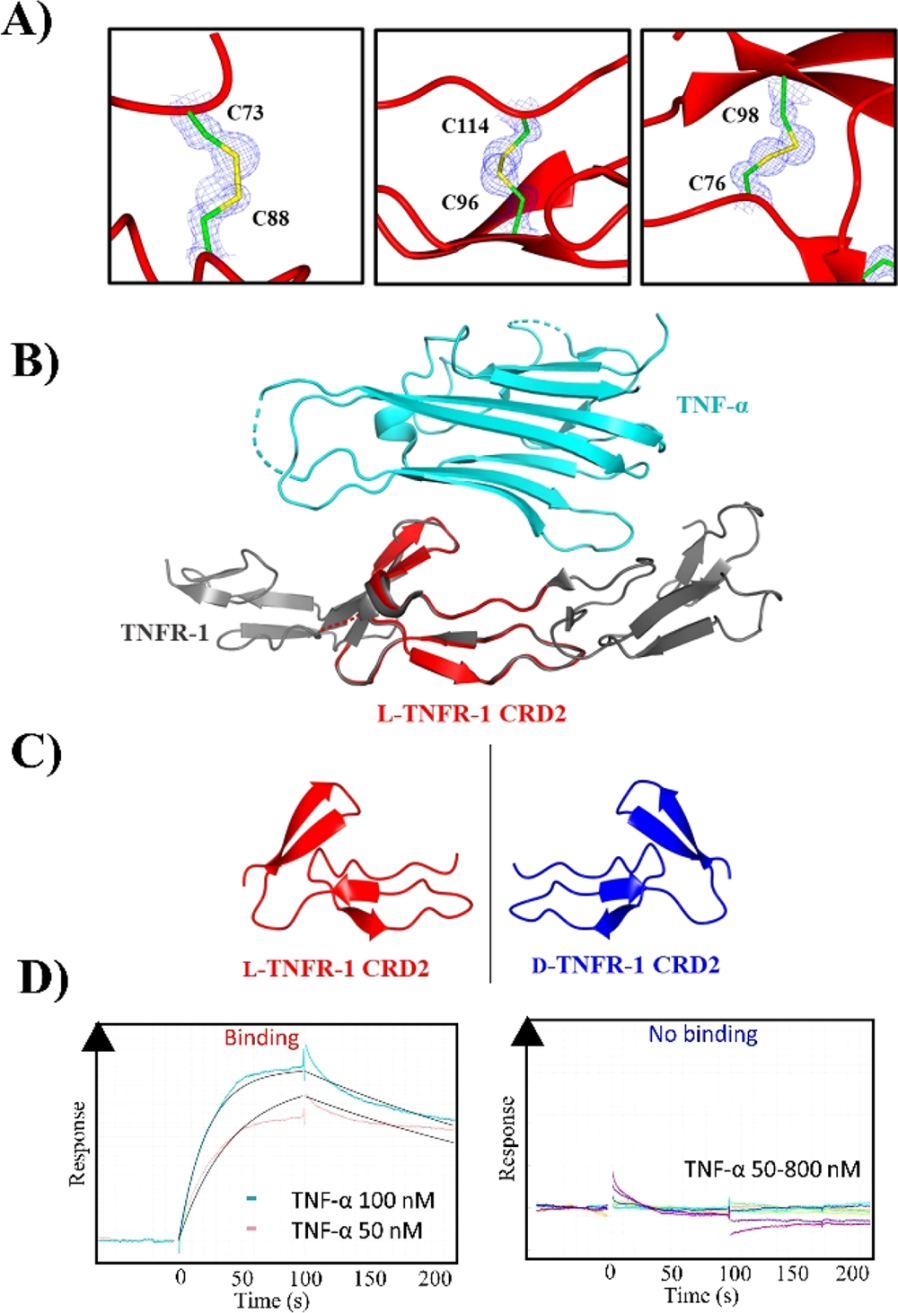
(A) σA-weighted 2*F*_*O*_–*F*_*C*_ omit electron density map of crystallized TNFR-1 CRD2 contoured at 1σ (0.46 electrons per Å^3^) showing the correct disulfide bond pairings (PDB: 8P6Q), (B) overlay of l-TNFR-1 CRD2 (PDB: 8P6Q) with TNFR-1/TNF-α structure (PDB: 7KPB) reveals retention of the native binding conformation (rmsd Cα = 0.36 Å), (C) mirror image folding of l- and d-TNFR-1 CRD2 observed in the racemic crystal, and (D) TNF-α binding to l-TNFR-1 CRD2 at 50–100 nM (left) and to d-TNFR-1 CRD at 50–800 nM (right). 1:1 Langmuir fit (left) is shown in black.

To validate the proper folding of TNFR-1 CRD2, we originally investigated the protein by circular dichroism spectroscopy. However, a low signal was obtained, likely due to a relatively low molar ellipticity of the short β sheet (Figure S2.1). Subsequently, we conducted racemic protein crystallography to analyze the structure of TNFR-1 CRD2.^4^ This technique involves crystallizing small proteins from a racemic protein mixture to gain prompt access of high-resolution structural data.^15d,17^ Here, enantiomeric TNFR-1 CRD2, prepared entirely from d-amino acids (d-TNFR-1 CRD2), was synthesized following the protocol developed above with an overall yield of 16%. The racemic TNFR-1 CRD2 mixture was subjected to sparse matrix crystallization screening, and the best crystal-forming condition was identified to contain 25 mg/mL of protein racemate, 1.5 M sodium chloride, and 10% v/v ethanol. The crystal structure was solved at 1.4 Å resolution (Table S2.1), confirming the correct disulfide bond patterns with well-defined electron density (Figure 3A). The TNFR-1 CRD2 structure aligned well with that of the entire receptor TNFR-1 in complex with TNF-α,^11c^ showing a backbone root-mean-square deviation (RMSD) of 0.36 Å across the cytokine binding interface (Figure 3B). Furthermore, the β sheet secondary structures observed in the original TNFR-1:TNF-α complex (PDB: 7KP7)^11c^ are conserved in the l-TNFR-1 CRD2 miniprotein, and are accurately reflected in the mirror image d-TNFR-1 CRD2 (Figure S2.1).

Derivatives of l- and d-TNFR-1 CRD2 were subsequently prepared by the addition of a C-terminal biotinylated linker. These proteins were immobilized onto streptavidin-coated grating coupled interferometry (GCI) chips to test their ability to recognize the native TNFR-1 ligand. The binding experiments with human TNF-α (Peprotech) showed clear binding to l-TNFR-1 CRD2 (*K*_D_ ≈ 7 nM), with no binding to the d-counterpart (Figure 3D). Consequently, this indicated that the TNFR-1 CRD2 is structurally conserved, and its binding to TNF-α suggested that it can serve as a receptor mimic for further investigations. While terminal cysteine-rich domains have been prepared in the literature,^7b^ ligand binding often takes place at the central portions of the extracellular protein domain.^11b,11c,18^ To the best of our knowledge, this is the first example in which the central binding portion of an extracellular CRD was chemically prepared and subsequently folded into its native conformation.

### Application of d-TNFR-1 CRD2 as a Bait Molecule for d-peptide Inhibitor Discovery

The receptor mimic was subjected to mirror-image molecular screening as a means of seeking ligands that inhibit TNF-α binding. Peptides comprised entirely of d-amino acids (d-peptides) are attractive therapeutic candidates, as they are typically considered to be more resilient to proteolytic degradation and hence nonimmunogenic.^3,19^ A d-peptide inhibitor could be potentially tested as a candidate against TNFR-1-related inflammatory diseases localized in the protease-rich gastrointestinal tract.^10b,20^ Consequently, the d-TNFR-1 CRD2 serves as a receptor mimic in mirror-image phage display (MIPD), a molecular screening technique that exploits the concept of reflection symmetry for the discovery of peptide ligands in opposite chirality.^5b^ To conduct the experiment, d-TNFR-1 CRD2 was biotinylated at the C-terminus, immobilized onto streptavidin-coated beads, and subjected to phage display. Subsequently, bacteriophage libraries^21^ consisted of the sequence format CX_9_C where X could be any canonical amino acid and terminal cysteine residues used for cyclization were tested. Following three rounds of biopanning, the peptide sequence CFHCVWLGMEC was found to be enriched (Table S3.1) and next-generation sequencing showed that the WLG motif was relatively conserved (Table S3.2). However, the presence of a cysteine residue at position four suggested alternative disulfide bond formation patterns. This was confirmed by an additional phage display experiment, in which the WLG motif was fixed in the format CX_4_WLGX_2_C. Following three rounds of biopanning, the most abundant peptide sequence was found to be CFHCIWLGDEC with the re-emergence of cysteine at position 4 (Tables S3.3 and S3.4, Figure S3.1).

The inclusion of Cys4 suggested that there are three possible isomers in which the disulfide bond could form between residues 1 and 4, 1 and 11, or 4 and 11 (Figure S4.1). To isolate the most active conformer, the fully reduced peptide was oxidized in the presence of cystine or glutathione disulfide (Figure S4.2). These conformers were isolated by HPLC for analysis by chymotrypsin digestion and LCMS analysis as well as binding to d-TNFR-1 CRD2 (Figures S4.3-S4.6). The active conformer, herein referred to as the TNFR-1 CRD2 peptide binder (TCPB), was found to contain an intramolecular disulfide bond between residues 4–11, and was cysteinylated at position 1 (Figure S4.7). To further characterize the TCPB, an orthogonal cysteine protection strategy using cysteine acetamidomethyl groups was implemented, coupled with solid phase deprotection and oxidation steps with iodine to yield the target conformer (Figures S4.8 and 4.9).

**Figure 4:**
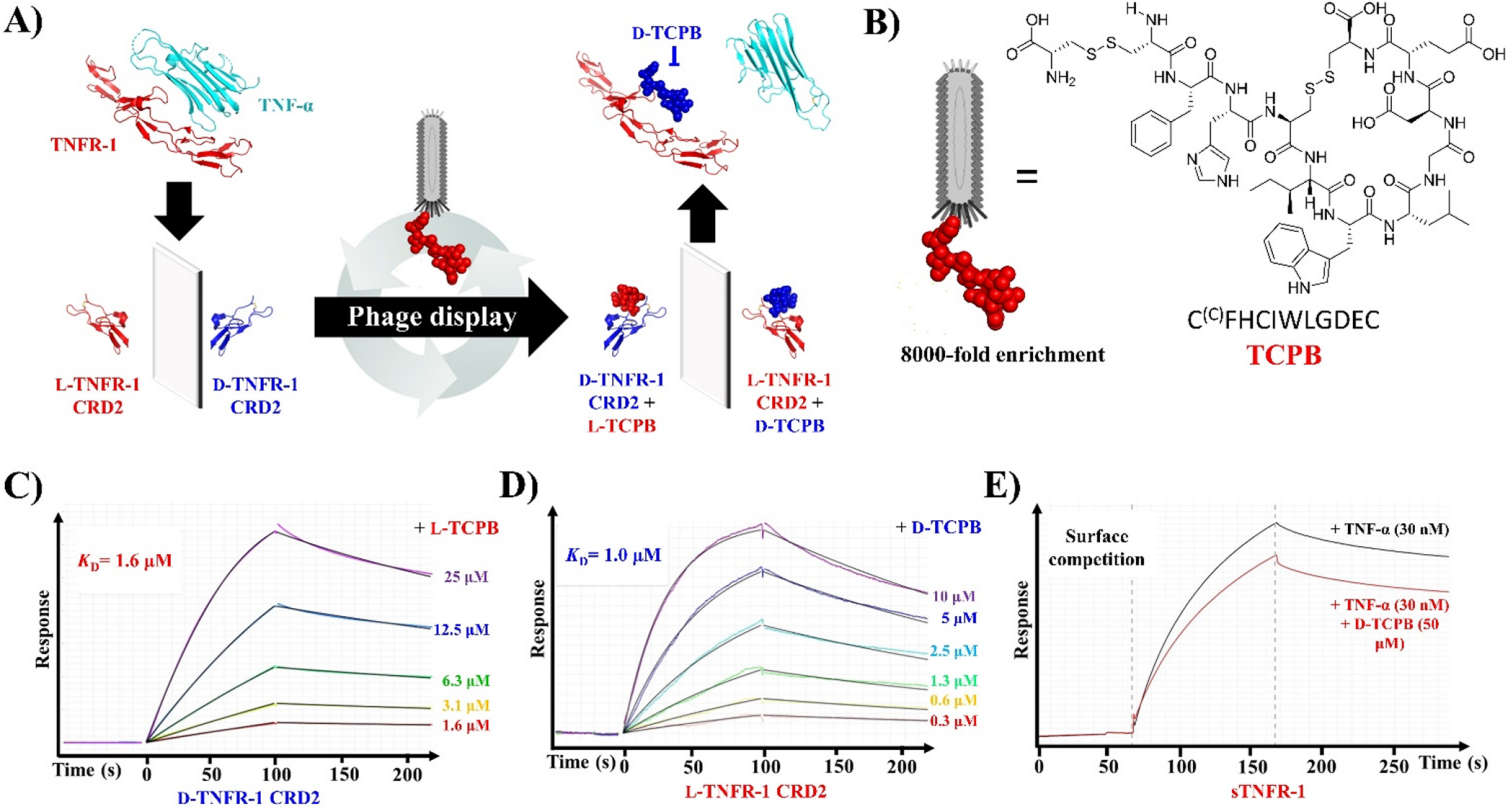
(A) Schematic overview of utilizing d-TNFR-1 CRD2 as a target in mirror image phage display for the discovery of d-peptide inhibitors. (B) Confirmed identify of the enriched peptide from phage display (TCPB). (C–E) Grating coupled interferometry (GCI) sensorgrams showing: (C) binding kinetics of l-TCPB to immobilized d-TNFR-1 CRD2, (D) binding kinetics of d-TCPB to immobilized l-TNFR-1 CRD2, and (E) TNF-α binding to immobilized sTNFR-1 with and without d-TCPB inhibitor.

Full analysis of the TCPB binding kinetics was conducted by GCI using serial 2-fold dilutions of concentration and demonstrated a binding affinity (*K*_D_) to d-TNFR-1 CRD2 of 1.6 μM (Figure 4C). The d-enantiomer of the candidate peptide binder (d-TCPB) also demonstrated reciprocal binding to the receptor mimic l-TNFR-1 CRD2 (*K*_D_ = 1.0 μM) (Figure 4D). Subsequently, d-TCPB was tested for binding to the full soluble extracellular portion of TNFR-1 (sTNFR-1, Peprotech). The binding kinetics parameters could not be obtained due to weak binding between d-TCPB and the sTNFR-1 (Figure S5.1). However, when TNF-α was flowed over immobilized sTNFR-1 in the presence of the d-TCPB, there is an observable reduction of TNF-α binding compared with the control without inhibitor, indicating competition for sTNFR-1 binding (Figure 4E).

The reduced affinity of the d-TCPB peptide for sTNFR-1 compared to the isolated CRD2 could be attributed to various factors, including steric clashes with residues outside of CRD2, interactions with the CRD2 N-terminus or interactions with neighboring charged residues on sTNFR-1 that affect long-range electrostatic associations or create electrostatic repulsion. Nevertheless, d-TCPB, serving as the first d-peptide lead, will be subjected to further refinement. Alternatively, other d-ligands can be isolated by preparing CRD2 capped at the N- and/or C-terminus or immobilized by different approaches. Subsequently, the new d-peptide lead will be tested directly for inhibition of TNFR-1/TNF-α using FRET-based (AlphaScreenTM) or ELISA in vitro assays.

## Conclusion

Here, we present a simple approach for preparing a miniprotein that can mimic the central cytokine binding segment of the tumor necrosis factor receptor 1 (TNFR-1 CRD2). Analysis by racemic protein crystallography and surface biophysical experiments demonstrated that the conformation of the receptor was retained. With a synthetically accessible TNFR-1 mimic available, we investigated its application in mirror image phage display and identified a d-peptide ligand for TNFR-1 CRD2. In clinical development, there are only a few d-peptides and l-RNA aptamers that can target naturally existing l-small proteins^22^ or -peptide epitopes,^8b,23^ most likely because preparation of d-protein targets is rather difficult. A simple route to d-TNFR-1 CRD2 may facilitate the discovery of ultrastable, chirally inverted molecules targeting TNFR-1, which can be potentially applied in the studies related to inflammatory diseases.^1a,10b,10c,24^ For example, d-peptide ligands targeting the TNFR-1 may be coupled with a chemical reporter, such as florescent- or radiolabeling,^25^ and utilized for the imaging/detection of TNFR-1 distributions.^26^ The TNFR-1 CRD2 synthesis scheme may also find uses in other biophysical and -physiological studies such as the investigation of specific post-translational modifications^27^ or the development of novel TNF-α biosensors.^28^ Indeed, TNF-α is an important biomarker in numerous inflammatory diseases,^29^ and synthetically accessible binding agents have demonstrated numerous advances in accessible biosensor technologies, such as electrochemical or fluorescent sensors.^30^ Finally, this study illustrated that synthesis of a central cysteine-rich domain is a feasible approach and may be applicable to the studies of other medically relevant receptors.^1d,31^

## Methods

### Peptide Synthesis

Peptides were prepared by automated SPPS and purified by preparative HPLC (see Supporting Information S6 for details). Isolated peptides were analyzed by HPLC-MS using a reversed-phase C18 column (ACE, 2.1 mm × 100 mm, 110 Å, 3 μm) held at 40 °C. A 5–70% gradient of acetonitrile (+0.1% TFA) in water (+0.1% TFA) was applied over 30 min, and analyte was detected using a UV detector at 210 and 280 nm and positive electrospray ionization mass spectrometry (ESI+ MS). Purity of all peptides and proteins is ≥95% as determined by the relative integration of the target peak in the chromatogram recorded at 210 nm.

### One-Pot Chemical Ligation and Refolding of TNFR-1 CRD2

Peptide hydrazide (Ser72-Ser87) (12.5 μmol), prepared by SPPS (see Supporting Information), was dissolved in 5 mL of 0.1 M sodium phosphate buffer containing 6 M Gn·HCl (pH 3.0–3.1) in a 20 mL round bottomed flask. The peptide solution was placed in an ice-salt bath at −15 °C and gently agitated by magnetic stirring for 10 min. In a 10 mL round bottomed flask, the N-terminal cysteine peptide (Cys88-Asn116 or Cys88-Asn116-Gly117-Gly121-Lys(Biotin)122) (10 μmol), prepared by SPPS (see Supporting Information S6) was dissolved in 5 mL of 0.1 M sodium phosphate buffer containing 6 M Gn·HCl (pH 6.9–7.0). The peptide hydrazide was oxidized into the corresponding peptide azide by addition of 10 equiv. NaNO2 (aq. 0.5 M) and gently stirred at −15 °C for 20 min.^32^ To convert the peptide azide to the thioester, trifluoroethanethiol was added (2% v/v), the solution was removed from the ice-salt bath, and the pH was adjusted to 5.0 at room temperature. Thioester conversion was allowed to proceed for 10 min, followed by addition of the N-terminal cysteine peptide (10 μmol in 5 mL of 0.1 M phosphate, 6 M Gn·HCl, pH 6.9–7.0). The pH of the ligation mixture was adjusted to 6.8–6.9 and an additional quantity of trifluoroethanethiol was added (1% v/v, final concentration 2% v/v). The flask was then placed in a heated water bath (37 °C) and stirred for 4 h. Reaction completion was confirmed by LCMS, by taking 10 μL of the reaction mixture and quenching with 80 μL of phosphate buffer (pH 3.0) and 10 μL of TCEP (0.1 M). Following ligation completion, excess trifluoroethanthiol was removed by purging with argon for 2 h at 37 °C.^16^ The reaction mixture (containing reduced ligation product) was transferred to a 2 L round bottomed flask, cooled to room temperature, and diluted with 120 mL of buffer (0.1 M phosphate, 6 M Gn·HCl, pH 6.5). In a 250 mL conical flask, glutathione (120 mM) and glutathione disulfide (12 mM) were dissolved in 130 mL of buffer (0.1 M phosphate, 6 M Gn·HCl, pH 6.5)). The buffer containing glutathione/glutathione disulfide was added to the diluted ligation reaction mixture (final ligation product conc. = 0.5 mg/mL) and stirred for 10 min at room temperature. The reaction mixture was then diluted 5-fold with phosphate buffer (0.1 M phosphate, pH 6.5) and stirred for 4 days at room temperature under an argon balloon. The folding reaction was monitored by LCMS, with the folded product indicated by a sharp peak with upfield retention and a molecular mass loss of 6 Da. The reaction mixture was filtered through a sintered glass funnel, and the folded protein was isolated by preparative HPLC (Final isolated yields l-TNFR-1 CRD2, 17%, 9.1 mg; d-TNFR-1 CRD2, 15%, 8.0 mg; d-TNFR-1 CRD2(biotin) at 1/10th scale (1.0 μmol), 14%, 0.84 mg; l-TNFR-1 CRD2 (biotin) at 1/10th scale (1.0 μmol), 13%, 0.78 mg).

### Racemic Protein Crystallography

l-TNFR-1 CRD2 and d-TNFR-1 CRD2 were dissolved in water to a final concentration of 25 mg/mL, as determined by UV absorbance at 280 nm.^33^ The peptide solutions were mixed in a 1:1 ratio to yield a 25 mg/mL racemate of dl-TNFR-1 CRD2. Half of the solution was diluted 2-fold with water to yield 12.5 mg/mL dl-TNFR-1 CRD2. The two racemate concentrations were subject to sparse-matrix crystallization screening using a Crystal Screen HT (HR2–130 from Hampton research). 50 μL of each precipitant condition was added into the wells of a SWISSCI 96-well plate. The two racemate concentrations were each mixed 1:1 with the precipitant in a 0.5 μL sitting drop, yielding 192 crystallization drops. The best TNFR-1 CRD2 crystals were formed in the sitting drop made with 0.25 μL of 25 mg/mL dl-TNFR-1 CRD2 and 0.25 μL of precipitant composed of 1.5 M sodium chloride and 10% v/v ethanol at pH 8.5. The single, block-shaped crystals were fished from the sitting drop and dipped into cryoprotectant (2.0 M Li_2_SO_4_) before flash-frozen in liquid nitrogen. X-ray diffraction data were collected at the Diamond Light Source synchrotron with beamline I03 using a Dectris Eiger2 XE 16M detector. The collected data sets were processed with Xia2, and data scaling performed with Aimless.^34^ The crystal space groups for data reductions were validated using Zandua.^35^ The structure of the l-TNRCD2 molecule in the protein crystal was first solved through molecular replacement in MOLREP, using residues 72–116 of the TNFR-1 X-ray crystal structure PDB: 1EXT as a search model.^36^ This led to the calculated phases delivering a clear electron density for the d-TNFR-1 CRD2 protein molecules. The structure of d-TNFR-1 CRD2, and the remaining waters and solute, was then built through iterative rounds of manual model building using COOT^37^ and anisotropic B-factor refinement via REFMAC.^38^

Data collection and refinement statistics are available in the Supporting Information (Table S2.1), and the refined model of racemic TNFR-1 CRD2 has been deposited in the Protein Data Bank with the PDB code 8P6Q.

### Phage Display

Briefly, a library of peptide sequences with nine randomized amino acid positions flanked by two cysteines (CX_9_C) were displayed on the M13 phages, where X is any amino acid encoded by NNK. The peptides were linked to the gene-3 coat protein (pIII) via a trialanine (Ala-Ala-Ala) spacer. Gel purified DNA encoding the libraries were ligated into the pCantab 5E phagemid vector, and transformed into chemically competent *E. coli* TG1. 20-fold excess of helper phage M13KO7 was used during rescue and amplification of the bacteriophages. The target protein (d-TNFR-1 CRD2) containing C-terminal GsGsGK(biotin) was immobilized (3 μg) onto streptavidin- or neutravidin-coated beads, alternating between panning rounds to reduce enrichment of nonspecific binders. Phage enrichment factor for each round is defined as the amount of phage eluted from the target well containing d- TNFR-1 CRD2 divided by the amount of phage eluted from the target well without immobilized target (Tables S3.1 and S3.3). For enriched sequences, see Tables S3.2 and 3.4.

### Surface Biophysical Experiments

Grating-coupled interferometry (GCI) experiments were performed on a Creoptix WAVEsystem using 4PCH-STA WAVE sensor chips (polycarboxylate surface, streptavidin coated) or 4PCH WAVE sensor chips (polycarboxylate surface) as indicated. Chips were first conditioned with borate buffer (0.1 M sodium borate, pH 9, 1 M Sodium chloride).

For binding of TNF-α and TCPB peptides to TNFR-1 CRD2, the TNFR-1 CRD2 containing the C-terminal flexible biotinylated linker (100 μg/mL) in HBS EP buffer (10 mM HEPES, 150 mM sodium chloride, 3 mM EDTA, 0.05% surfactant P20) was immobilized onto the 4PCH-STA sensor chip by injection over the sensor surface at a 10 μL/min flow rate, followed by passivation of the surface with blank HBS EP buffer. Final immobilization densities of 2300 pg/mm^2^ of d- TNFR-1 CRD (biotin) and 1000 pg/mm^2^l-TNFR-1 CRD2(biotin) were obtained. The subsequent GCI experiments were conducted in HBS EP running buffer.

TNF-α binding to the d-TNFR-1 CRD2(biotin) and l-TNFR-1 CRD2(biotin) was determined by preparing stocks of human TNF-α (Peprotech, 300–01A) in HBS-EP buffer at a 50–800 nM concentration. The cytokine solutions were passed over the immobilized d- and l-TNFR-1 CRD2 flow cells and a blank reference flow cell at a flow rate of 30 μL/min for a 100 s total injection time, followed by a 120 s dissociation phase with the HBS EP buffer. The surface was regenerated with 10 mM Glycine·HCl (pH 2) during each cycle. GCI sensorgrams are shown in Figure 3D.

l-/d-TCPB binding to the TNFR-1 CRD2 was conducted as above, using serial 2-fold dilutions (as indicated in legend, Figure 4C and D) in HBS-EP buffer. Data were fitted with the one-to-one Langmuir binding model and binding affinity analysis conducted in the Creoptix Wave control software.

For d-TCPB binding to sTNFR-1, and the TNF-α competition assay, human sTNFRI (animal free, Peprotech, AF-310-07) was immobilized onto a 4PCH chip via amine coupling. The polycarboxylate surface was activated by flowing a 1:1 mixture of 0.4 M EDC in H_2_O and 0.1 M NHS in H_2_O over all four flow channels at a rate of 10 μL/min sTNFR-1 (10 μg/mL) in 10 mM sodium acetate, pH 4.0, was then immobilized covalently to the sample cell at a flow of 10 μL/min until a surface density of 500 pg/mm^2^ was achieved. The sample and reference flow cells were subsequently blocked by injection of 1 M ethanolamine·HCl, pH 8.5, at a flow rate of 10 μL/min. d-TCPB binding to the TNFR-1 was conducted as above, using serial 2-fold dilutions (as indicated in legend, Figure S5.1) in HBS-EP buffer (containing 0.005% P20). The surface competition assay was conducted by flowing TNF-α (30 nM) over the sTNFR-1 sensor chip, either alone or with added d-TCPB (50 μM). The GCI sensorgrams are shown in Figure 4E.

In all experiments, blank injections were used for double referencing and a DMSO calibration curve was used for bulk correction.

For further experimental details and peptide/protein LCMS data, see the Supporting Information.

## Data Availability

Model coordinates and structure factors for the racemic protein crystal structure of TNFR-1 CRD2 reported in this work has been deposited in the Protein Data Bank under the accession code 8P6Q

## Supporting Information Available

Supplementary figures and methods, HPLC and mass spectrometry data (PDF)

## Abbreviations

TNFR-1: tumor necrosis factor receptor 1
TNF-α: tumor necrosis factor α
CRD: cysteine-rich domain
SPPS: solid-phase peptide synthesis
MPAA: 4-mercaptophenylacetic acid
TFET: trifluoroethanthiol
MIPD: mirror-image phage display
TCPB: TNFR-1 CRD2 peptide binder
GCI: grating-coupled interferometry
