# Supplementary material for: Deciphering the Synthetic and Refolding Strategy of a Cysteine-Rich Domain in the Tumor Necrosis Factor Receptor (TNF-R) for Racemic Crystallography Analysis and d-Peptide Ligand Discovery
Source: ACS Bio Med Chem Au. 2023 Dec 11;4(1):68–76. doi: 10.1021/acsbiomedchemau.3c00060 (PMC10885103; doi:10.1021/acsbiomedchemau.3c00060)
Supplement: Supplementary file 1 — bg3c00060_si_001.pdf [file bg3c00060_si_001.pdf]

Supporting information

Deciphering the Synthetic and Refolding Strategy of a Cysteine-Rich Domain in the Tumor Necrosis Factor Receptor (TNF-R) for Racemic Crystallography analysis and D-Peptide Ligand Discovery

Alexander J. Lander,[§] Yifu Kong,[†] Yi Jin,*[‡] Chuanliu Wu*[†] and Louis Y.P. Luk.*[§]

AUTHOR ADDRESS

[§]School of Chemistry, Cardiff University, Main Building, Park Place, Cardiff, CF10 3AT (UK). Email: lukly@cardiff.ac.uk

[†]Department of Chemistry, College of Chemistry and Chemical Engineering, The MOE Key Laboratory of Spectrochemical Analysis and Instrumentation, State Key Laboratory of Physical Chemistry of Solid Surfaces, Xiamen University, Fujian Province, 361005 (China). Email: chlwu@xmu.edu.cn

[‡]Manchester Institute of Biotechnology, University of Manchester, Manchester, M1 7DN (UK). Email: yi.jin@manchester.ac.uk

Table of contents

S1. Optimization of TNFR-1 CRD2 refolding 4
S2. Racemic protein crystallography of TNFR-1 CRD2 6
S3. Phage display screening against D-TNFR-1 CRD2 9
S4. Identification of active binding peptide conformer 13
S5. Binding of the D-TCPB peptide to sTNFR-1 20
S6. Experimental procedures 21
S7. Peptide and protein LCMS data..24
References..39

S1. Optimization of TNFR-1 CRD2 refolding

Optimization of oxidative reagents

Reduced TNFR-2 CRD2 (0.5 mg/mL – measured by UV absorbance[1]) was dissolved in 200 μL of denaturation buffer in a 2 mL centrifuge tube (**i**. 6 M Gn·HCl, 0.25 M $NaHCO_3$, 6 mM glutathione disulfide, 60 mM glutathione, pH 8.5 ; **ii.** 6 M Gn·HCl, 0.25 M $NaHCO_3$, 10% DMSO, pH 8.5 ; **iii.** 6 M Gn·HCl, 0.25 M $NaHCO_3$, pH 8.5 ; **iv**. 6 M Gn·HCl, 0.1 M Tris, 6 mM glutathione disulfide, 60 mM glutathione, pH 8.5). Each reaction mixture **i-iv** was diluted five-fold with 800 μL renaturation buffer (**i-iii** 0.25 M $NaHCO_3$, pH 8.5 ; **iv**. 0.1 M Tris, pH 8.5). Folding reactions proceeded at room temperature for 96 hours. 100 μL of reaction was removed, filtered through glass wool, and analyzed by LCMS. The refolding yield for each condition was estimated by the relative integration of the peak shifted up field by 1 min in the HPLC chromatogram at 210 nm (Table S1). Condition **iv** produced the highest yield (9%) and moved onto the next round of optimization below.

Optimization of buffer pH and temperature

Reduced TNFR-1 CRD2 (0.5 mg/mL – measured by UV absorbance[1]) was dissolved in 200 μL of denaturation buffer in a 2 mL centrifuge tube (**i** & **iii**. 6 M Gn·HCl, 0.1 M Tris, 6 mM glutathione disulfide, 60 mM glutathione, pH 8.5 ; **ii** & **iv.** 6 M Gn·HCl, 0.1 M phosphate, 6 mM glutathione disulfide, 60 mM glutathione, pH 6.5). Each reaction mixture **i-iv** was diluted five-fold with 800 μL renaturation buffer (**i** & **iii.** 0.1 M Tris, pH 8.5 ; **ii** & **iv.** 0.1 M phosphate, pH 6.5). Folding reactions proceeded for 96 hours, either at room temperature (**i-ii**) or at 4 °C (**iii-iv**). 100 μL of reaction was removed, filtered through glass wool, and analyzed by LCMS. The refolding yield for each condition was estimated by the relative integration of the peak shifted up field by 1 min in the HPLC chromatogram at 210 nm (Table S7.2). Condition **ii** produced the highest yield (33%) and moved onto the next round of optimization below.

Optimization of reactant concentrations

Reduced TNFR-1 CRD2 (0.5 mg/mL **i-ii**, or 2 mg/mL **iii-v** – measured by UV absorbance[1]) was dissolved in 200 μL of denaturation buffer in a 2 mL centrifuge tube (6 M Gn·HCl, 0.1 M phosphate, pH 6.5 containing ; **i**, **iii** & **v.** 6 mM glutathione disulfide, 120 mM glutathione ; **ii** & **iv.** 25 mM glutathione disulfide, 50 mM glutathione). Each reaction mixture **i-v** was diluted five-fold with 800 μL renaturation buffer (0.1 M phosphate, pH 6.5). Folding reactions proceeded for 96 hours at room temperature. 100 μL of reaction was removed, filtered through glass wool, and analyzed by LCMS. The refolding yield for each

condition was estimated by the relative integration of the peak shifted up field by 1 min in the HPLC chromatogram at 210 nm (Table S7.2). Neither condition produced in improvement in refolding yield (<21%). Higher TNRCD2 concentrations were desirable as this would result in smaller reaction volumes during scale-up preparations. However, folding at 0.4 mg/mL (**iii-v**) gave significantly reduced yield (5-13%). Therefore, the best condition (**ii**) from the previous round was used for TNRCD2 refolding.

Table S1.1: Conditions used during screening of TNFR-1 CRD2 refolding. First screening oxidation method (GSSG/GSH best), then buffer pH and temperature (0.1 M NaPi, pH 6.5, 25 ºC best), then finally reagent concentrations (all worse than previous). The conditions with the highest yield of folded TNFR-1 CRD2 are highlighted.

**Component**	**Oxidation method**				**Buffer pH and temperature**				**Concentrations**				
**Denaturation**	**i**	**ii**	**iii**	**iv**	**i**	ii	**iii**	**iv**	**i**	**ii**	**iii**	**iv**	**v**
GnHCl	6M	6M	6M	6M	6M	6M	6M	6M	6M	6M	6M	6M	6M
Oxidant	6mM GSSG	10% DMSO	atmos. O2	6mM GSSG	6mM GSSG	6mM GSSG	6mM GSSG	6mM GSSG	6mM GSSG	25mM GSSG	6mM GSSG	25mM GSSG	6mM GSSG
Reductant	60mM GSH	-	-	60mM GSH	60mM GSH	60mM GSH	60mM GSH	60mM GSH	120mM GSH	50mM GSH	120mM GSH	50mM GSH	120mM GSH
Buffer	-	-	-	0.1M Tris	0.1M Tris	0.1M NaPi	0.1M Tris	0.1M NaPi	0.1M NaPi	0.1M NaPi	0.1M NaPi	0.1M NaPi	0.1M NaPi
**Dilute x5 with**	0.25M NaHCO3	0.25M NaHCO3	0.25M NaHCO3	0.1M Tris	0.1M Tris	0.1M NaPi	0.1M Tris	0.1M NaPi	0.1M NaPi	0.1M NaPi	0.1M NaPi	0.1M NaPi	0.1M NaPi
pH	8.5	8.5	8.5	8.5	8.5	6.5	8.5	6.5	6.5	6.5	6.5	6.5	6.5
TNFR-1 CRD2 final conc. (mg/mL)	0.1	0.1	0.1	0.1	0.1	0.1	0.1	0.1	0.1	0.1	0.4	0.4	0.4
T (°C)	25	25	25	25	25	25	4	4	25	25	25	25	25
refolding % 96 hrs	7%	<2%	0	9%	17%	33%	0%	3%	15%	21%	7%	5%	13%

S2. Racemic protein crystallography of TNFR-1 CRD2

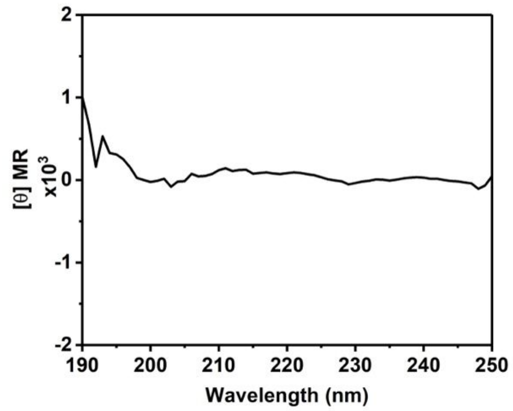

Figure S2.1: Circular dichroism spectrum of L-TNFR-1 CRD2 in ddH_2O (40 μM), recorded from 250 nm to 190 nm at 20 °C using an Applied Photophysics Chirascan CD spectrometer. Each measurement was performed in triplicate using a sample cell with a 0.1 mm path, 1 nm bandwidth and 0.5 s per point. CD spectra of the solvents were subtracted from the CD spectra of the proteins to eliminate interference. The units of ellipticity are expressed as the mean residue ellipticity ([θ]MR) in $deg \cdot cm^2 \cdot dmol^{-1} \cdot res^{-1}$.

Table S2.1: X-ray data collection, processing, and refinement statistics.

	**TNFR-1 CRD2**
**PDB accession**	8P6Q
**Data collection**	
**Space group**	$P12_12$
**Cell dimensions**	
**a, b, c (Å)**	20.4, 50.5, 46.2
**α, β, γ ()**	90.0, 92.9, 90.0
**Resolution (Å)**	34.06 – 1.40 (1.42 – 1.40)
**R_{sym} or R_{merge}**	0.090 (1.076)
**I / σI**	11.3 (1.0)

**Completeness (%)**	100 (100)
**Redundancy**	6.0 (5.7)
**Refinement**	
**Resolution (Å)**	34.06 – 1.40
**No. reflections**	18533 (943)
**R_{work} / R_{free}**	0.200 / 0.251
**No. atoms**	
**Protein**	793
**Ligand/ion**	35
**Water**	120
**B-factors**	
**Protein**	16.15
**Ligand/ion**	16.7
**Water**	27.58
**R.m.s deviations**	
**Bond lengths (Å)**	0.0090
**Bond angles ()**	1.662

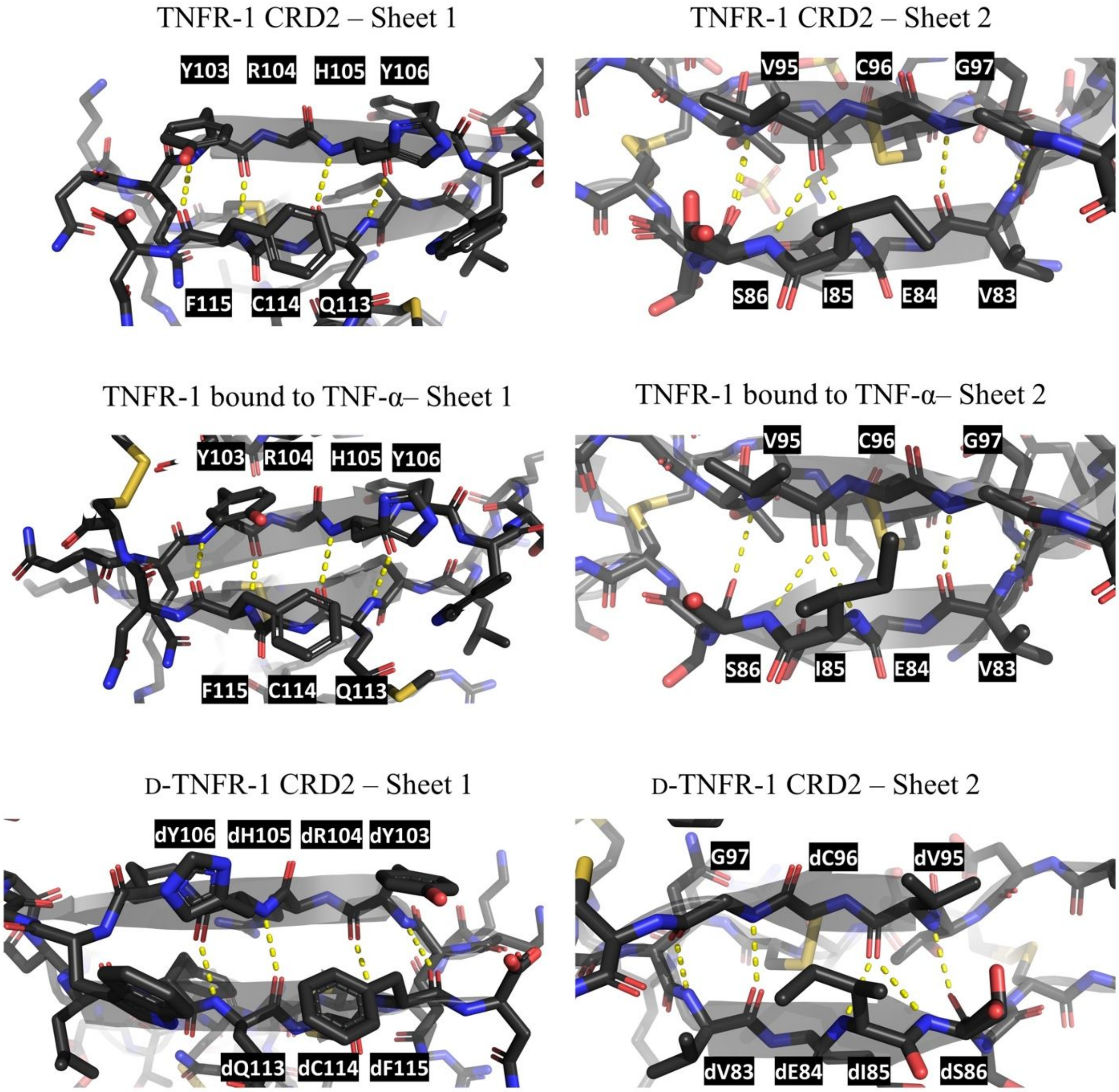

Figure S2.2: Beta-sheet secondary structures of L-TNFR-1 CRD2 (PDB: 8P6Q) are conserved with L-TNFR-1 in TNF- α-bound state (PDB: 7KP7), and accurately reflected in the mirror image D-TNFR-1 CRD2 (PDB: 8P6Q). Involving residues are indicated, and interchain H-bonds shown as yellow dashes.

S3. Phage display screening against D-TNFR-1 CRD2

Table S3.1: Results of bacteriophage biopanning experiment 1 with the sequence format CX_9C. Enrichment factor for each round is defined as the amount of phage eluted from the target well containing D-TNFR-1 CRD2 divided by the amount of phage eluted from the target well without immibilized target (streptavidin/neutravidin only).

Screening round number	Amount of immobilized D-TNFR-1 CRD2 (μg)	Amount of phage input (pfu)	Amount of phage in elution from target well (pfu)		Enrichment factor
1	3	4.96×10^{12}	Test	4.18×10^{6}	58
			Control	7.20×10^{4}	
2	3	2.85×10^{13}	Test	1.08×10^{7}	5.36
			Control	2.02×10^{6}	
3	3	7.00×10^{12}	Test	9.43×10^{7}	152.32
			Control	6.19×10^{5}	

Table S3.2: Next generation sequencing of enriched CX_9C bacteriophage library following screening experiment 1. A total of 20282 sequences were identified, with sequences in abundance >40 listed below, in decreasing order.

**Amino acid sequence**											
P1	P2	P3	P4	P5	P6	P7	P8	P9	P10	P11	**Abundance**
**C**	F	H	C	V	W	L	G	M	E	**C**	7590
**C**	W	H	V	A	W	L	G	E	G	**C**	630
**C**	I	T	V	L	P	G	I	V	V	**C**	556
**C**	Q	E	K	R	G	T	P	E	E	**C**	366
**C**	W	W	R	E	D	Q	Y	Q	Q	**C**	166
**C**	E	C	V	D	V	E	P	F	F	**C**	130
**C**	V	E	S	L	L	P	P	W	W	**C**	130
**C**	L	R	A	V	A	W	W	D	D	**C**	114
**C**	M	L	P	A	P	L	E	L	L	**C**	100
**C**	W	E	N	W	E	D	T	W	W	**C**	94
**C**	Y	A	D	Y	R	E	L	K	K	**C**	80
**C**	L	E	V	R	S	R	R	D	D	**C**	72
**C**	R	L	E	Y	L	P	F	V	V	**C**	64
**C**	V	V	V	V	P	G	R	I	I	**C**	54
**C**	R	L	K	L	E	L	A	S	S	**C**	52
**C**	N	V	L	R	E	G	R	S	S	**C**	52
**C**	W	A	R	E	S	P	R	K	K	**C**	50
**C**	I	L	P	G	P	L	E	L	L	**C**	50
**C**	E	L	F	G	P	L	W	S	S	**C**	48
**C**	P	P	A	P	E	R	T	P	P	**C**	46
**C**	V	E	P	W	E	G	K	I	I	**C**	42
**C**	F	H	V	V	W	L	G	G	E	**C**	40
**C**	W	E	V	I	W	R	E	G	W	**C**	40

Table S3.3: Results of bacteriophage biopanning experiment 2 with the sequence format CX_4WLGX_2C. Enrichment factor for each round is defined as the amount of phage eluted from the target well containing D-TNFR-1 CRD2 divided by the amount of phage eluted from the target well without immobilized target (streptavidin/neutravidin only).

Screening round number	Amount of immobilized D-TNFR-1 CRD2 (μg)	Amount of phage input (pfu)	Amount of phage in elution from target well (pfu)		Enrichment factor
1	3	2.00×10^{13}	Test	7.20×10^{6}	10
			Control	7.20×10^{5}	
2	3	7.50×10^{12}	Test	1.08×10^{7}	5.36
			Control	2.02×10^{6}	
3	3	2.75×10^{13}	Test	2.45×10^{10}	8292.68
			Control	2.95×10^{6}	

Table S3.4: Sequencing of enriched CX_4WLGX_2C bacteriophage library following screening experiment 2. A total of 22 monoclones were randomly selected, with sequences in listed below, in decreasing order of abundance.

Amino acid sequence											
P1	P2	P3	P4	P5	P6	P7	P8	P9	P10	P1<br>1	**Abundance**
**C**	F	H	C	I	**W**	**L**	**G**	D	E	**C**	4
**C**	Y	H	C	V	**W**	**L**	**G**	H	E	**C**	3
**C**	Y	H	I	I	**W**	**L**	**G**	D	E	**C**	2
**C**	F	H	C	I	**W**	**L**	**G**	P	E	**C**	2
**C**	Y	H	I	V	**W**	**L**	**G**	N	E	**C**	1
**C**	Y	H	C	V	**W**	**L**	**G**	T	E	**C**	1
**C**	Y	H	C	I	**W**	**L**	**G**	F	E	**C**	1
**C**	F	H	C	I	**W**	**L**	**G**	L	E	**C**	1
**C**	F	H	C	I	**W**	**L**	**G**	T	E	**C**	1
**C**	Y	E	V	I	**W**	**L**	**G**	H	E	**C**	1
**C**	F	H	C	I	**W**	**L**	**G**	N	E	**C**	1
**C**	F	H	C	I	**W**	**L**	**G**	S	E	**C**	1
**C**	F	H	I	I	**W**	**L**	**G**	N	E	**C**	1
**C**	F	H	C	I	**W**	**L**	**G**	F	E	**C**	1
**C**	M	W	C	E	**W**	**L**	**G**	E	D	**C**	1

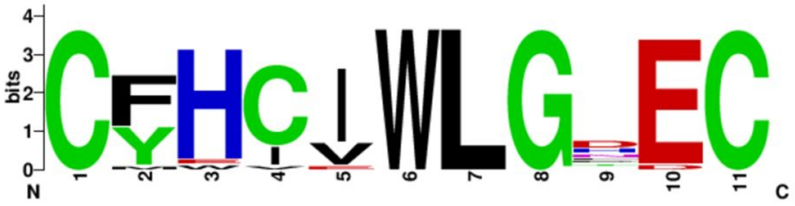

Figure S3.1: Sequence consensus of the enriched peptide library following the second biopanning experiment, using a CX_4WLGX_2C library format.

S4. Identification of active binding peptide conformer

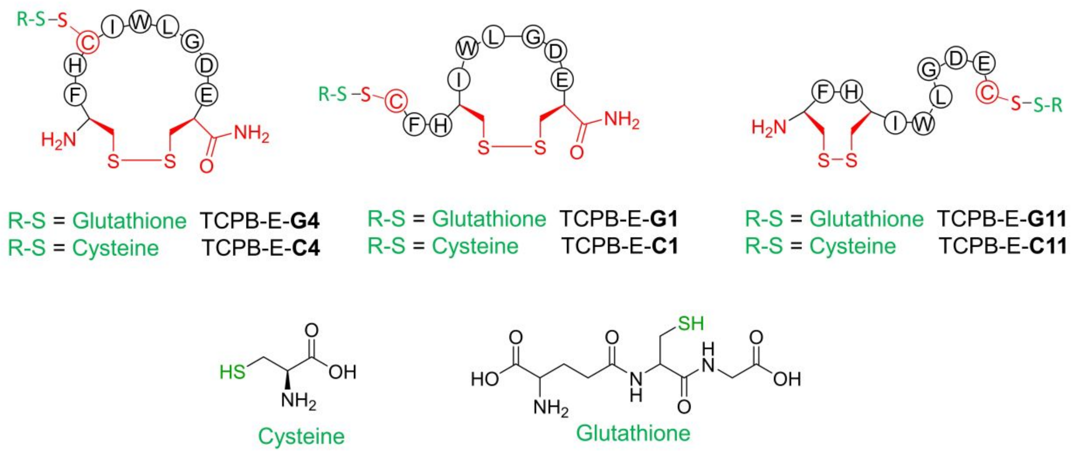

Figure S4.1: Six possible conformers of TCPB, oxidized with either glutathione (TCPB-E-G) or cysteine (TCPB-E-C).

TCPB solution phase oxidation

TCPB-E was prepared by automated SPPS (See S5) and isolated by preparative HPLC in fully reduced form. In a 10 mL round bottomed flask, the peptide (50 μM) was dissolved in 5 mL of buffer (0.1 M phosphate, pH 6) containing 0.5 mM of either cystine or glutathione. The reaction mixtures were stirred for six hours at room temperature. Reaction completion was confirmed by LCMS, by taking 10 μL of reaction mixture and diluting with 90 μL of distilled water. The two disulfide bonded products for each cysteine and glutathione mixture were isolated by semi-preparative HPLC. LCMS analysis is provided in Figure S3.2.

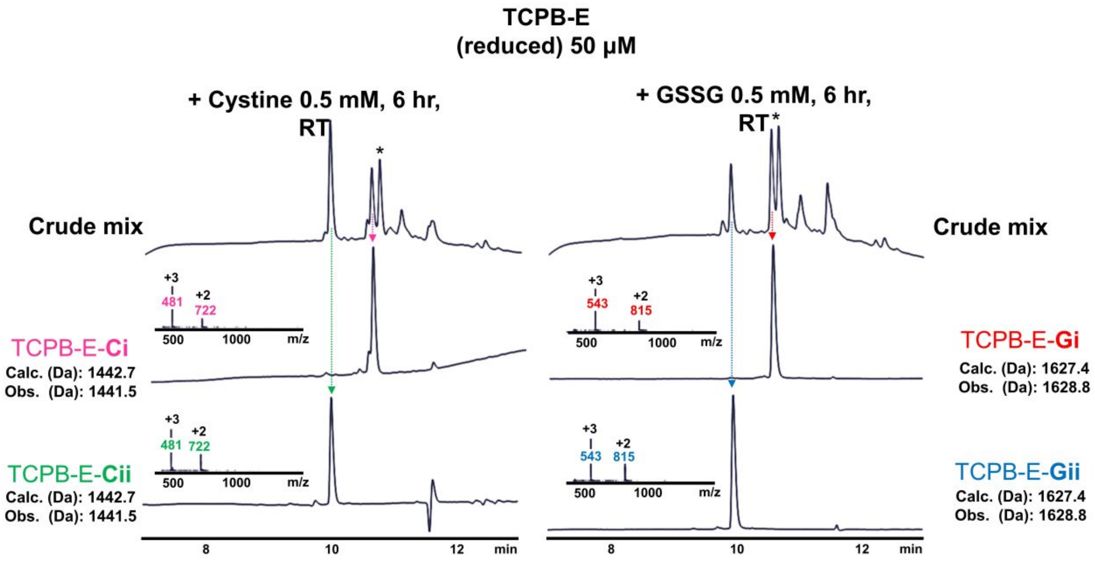

Figure S4.2: Solution-phase oxidation of TCPB-E with cystine (left) or glutathione disulfide (right) for deduction of active bacteriophage conformation. See below for identification of peaks by disulfide bond mapping experiments. Peak corresponding to dimerized product with three disulfide bonds indicate by asterisk (*).

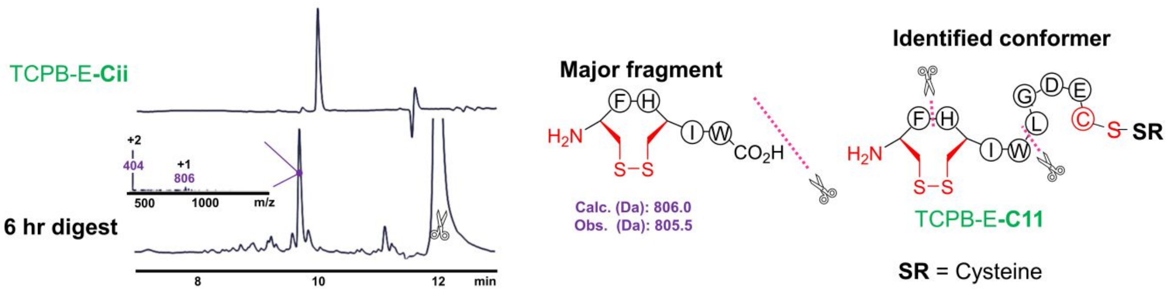

Figure S4.3: LCMS disulfide bond mapping of isolated TCPB-E peak **ii**, oxidized with cystine. Lyophilized peptide was digested with chymotypsin (0.1 mg/mL) in phosphate buffer (0.1 M, pH 6) at room temperature for 6 hours. HPLC trace of undigested peptide is shown in top left, and HPLC trace and corresponding ESI+ MS of major digested peak is shown in bottom left. Identity of the major digested fragment and the deduced, undigested conformer are shown to the right. No digestion was observed between Phe2 and His3 after 6 hours.

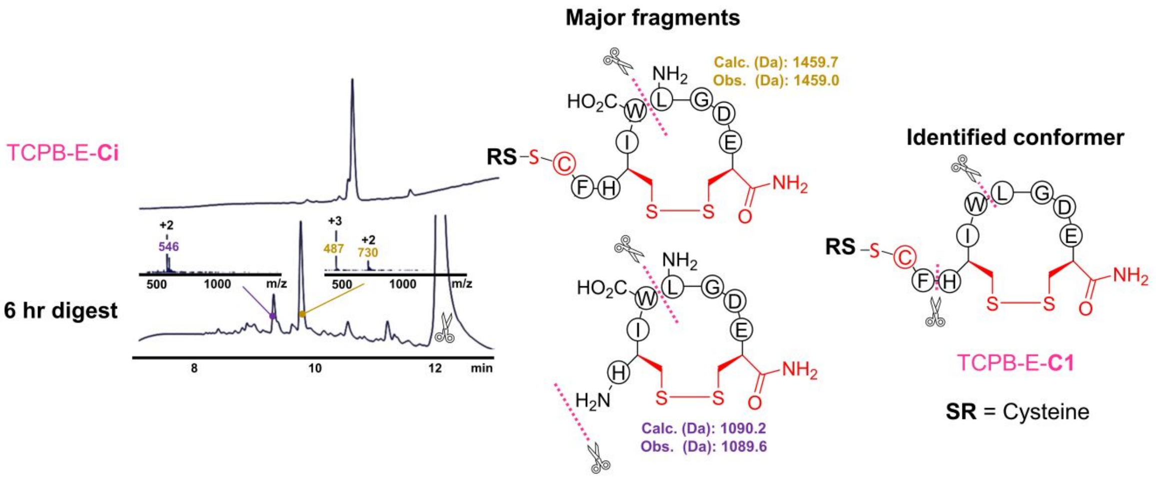

Figure S4.4: LCMS disulfide bond mapping of isolated TCPB-E peak **i**, oxidized with cystine. Lyophilized peptide was digested with chymotypsin (0.1 mg/mL) in phosphate buffer (0.1 M, pH 6) at room temperature for 6 hours. HPLC trace of undigested peptide is shown in top left, and HPLC trace and corresponding ESI+ MS of major digested peaks are shown in bottom left. Identity of the major digested fragments and the deduced, undigested conformer are shown to the right.

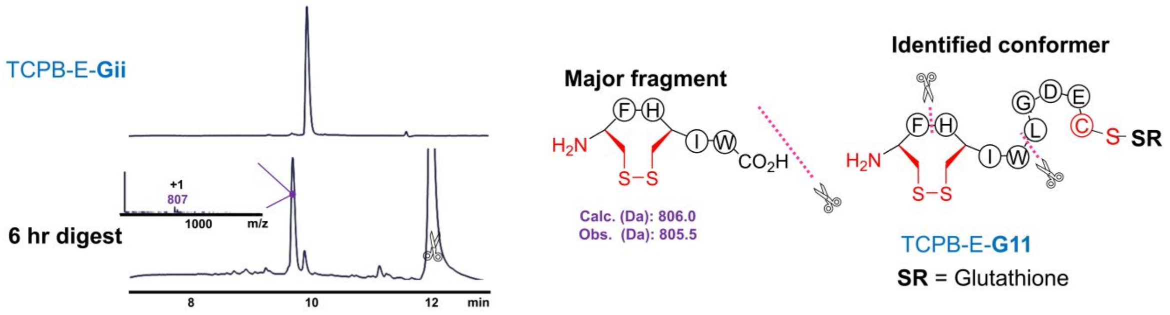

Figure S4.5: LCMS disulfide bond mapping of isolated TCPB-E peak **ii**, oxidized with glutathione disulfide. Lyophilized peptide was digested with chymotypsin (0.1 mg/mL) in phosphate buffer (0.1 M, pH 6) at room temperature for 6 hours. HPLC trace of undigested peptide is shown in top left, and HPLC trace and corresponding ESI+ MS of major digested peak is shown in bottom left. Identity of the major digested fragment and the deduced, undigested conformer are shown to the right. No digestion was observed between Phe2 and His3 after 6 hours.

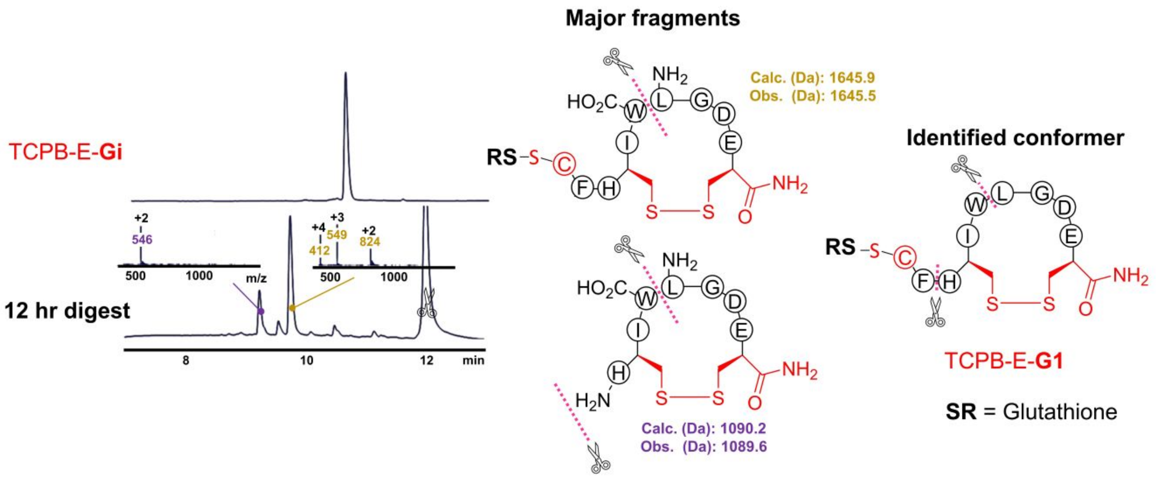

Figure S4.6: LCMS disulfide bond mapping of isolated TCPB-E peak **i**, oxidized with glutathione disulfide. Lyophilized peptide was digested with chymotypsin (0.1 mg/mL) in phosphate buffer (0.1 M, pH 6) at room temperature for 12 hours. HPLC trace of undigested peptide is shown in top left, and HPLC trace and corresponding ESI+ MS of major digested peaks are shown in bottom left. Identity of the major digested fragments and the deduced, undigested conformer are shown to the right.

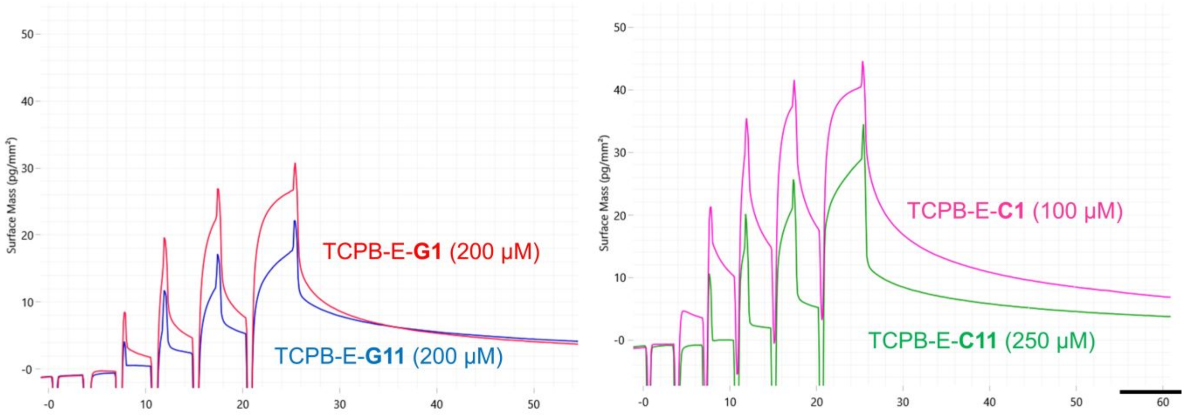

Figure S4.7: Grating-coupled interferometry (GCI) analysis of TCPB-E isomers binding to D-TNFR-1 CRD2. Repeated analyte pulses of increasing duration (RAPID) were passed over the sensor surface, with fixed concentrations of each peptide conformer shown above. Sensograms shown are blank subtracted (target flow cell - flow cell with no target). The highest sensor response with respect to concentration was observed for TCPB-E-**C1**.

TCPB solid-phase oxidation

To obtain high purity samples of the cyclic peptide conformer for kinetic analysis, and access the remaining conformer (disulfide 1-11), a solid-phase synthetic approach was devised. The procedure utilizes the differential reactivity of iodine towards orthogonally protected cysteine residues, based on a previously reported procedure in solution-phase.[2] The method was optimized here for application on the solid-phase. Briefly, in non-polar solvents, Iodine selectively removes Cys(Trt) groups and oxidizes cysteines to form disulfide bonds, with Cys(Acm) left intact. In the more polar DMF, Cys(Acm) is removed and enables formation of the second disulfide bond. This method circumvents the use of the commonly employed Cys(Mmt) and *N*-chlorosuccinimide, due to the high cost of the Fmoc-D-Cys(Mmt)-OH building block.

TCPB sequence was assembled by automated SPPS (See S5) on a low-loading PEG-PS rink amide resin (CEM, 0.19 mmol/g) to minimize unwanted intermolecular disulfide bond formation. For TCPB-E-**C1**, Fmoc-Cys(Trt)-OH was used at position 1, and Fmoc-Cys(Acm)-OH used at positions 4 and 11. For TCPB-E-**C4**, Fmoc-Cys(Trt)-OH was used at position 4, and Fmoc-Cys(Acm)-OH used at positions 1 and 11. First, the intermolecular disulfide bond with cysteine was formed. Boc-Cys(Trt)-OH (2 equiv., 60 mM) was dissolved in 50% TFE in DCM and added to the peptide resin. Then, an equal volume of Iodine (1 equiv., 30 mM) in 50% TFE in DCM was also added to the resin, and the slurry stirred for 10 mins at room temperature. The resin was washed with DCM (3 mL) and the reaction was repeated. The resin was washed once with DCM (3 mL) and three times with DMF (3 x 3 mL). For intramolecular disulfide bond formation, ten equivalents of Iodine in DMF (0.5 M) was added to the resin, and the slurry stirred for 60 mins at room temperature. The resin was then washed three times with DMF (3 × 3 mL), once with 1 M aq. ascorbic acid (3 mL), three times with water (3 × 3 mL) and three times with DMF (3 × 3 mL). The peptide was cleaved from the resin and isolated by preparative HPLC.

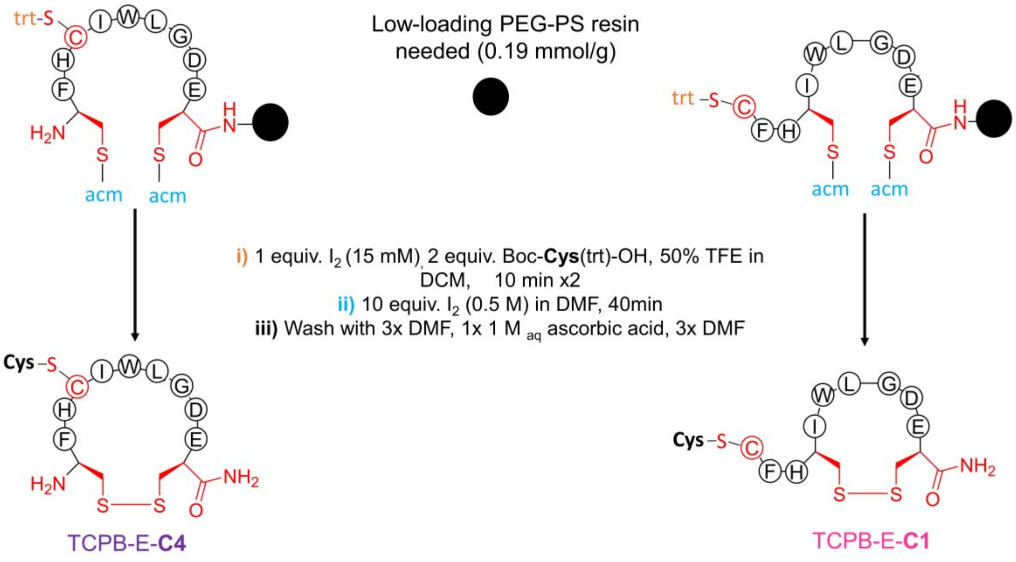

Figure S4.8: Illustrative representation of a solid-phase synthetic route to TCPB-E peptide conformers. An orthogonal protection scheme was implemented using cysteine acetamidomethyl (acm) or trityl (trt). Disulfide bond formation was controlled by the relative reactivity of Iodine towards protected cysteine residues in non-polar (trt-trt) and polar (acm-acm) solvents, based on a modified procedure.[2]

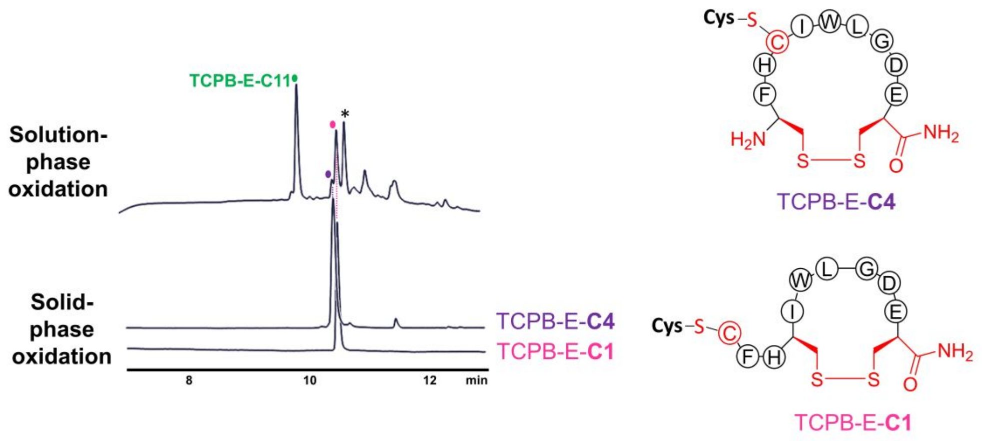

Figure S4.9: Comparison of HPLC retention times of TCPB-E-**C1** and -**C4** prepared on solid-phase with the solution-phase oxidation of TCPB-E with cystine, reinforcing the results of LCMS disulfide bond mapping. Small quantities of TCPB-E-**C4** appear to have been formed in solution-phase but was insufficient for isolation, likely suggesting that this conformer is thermodynamically unfavorable.

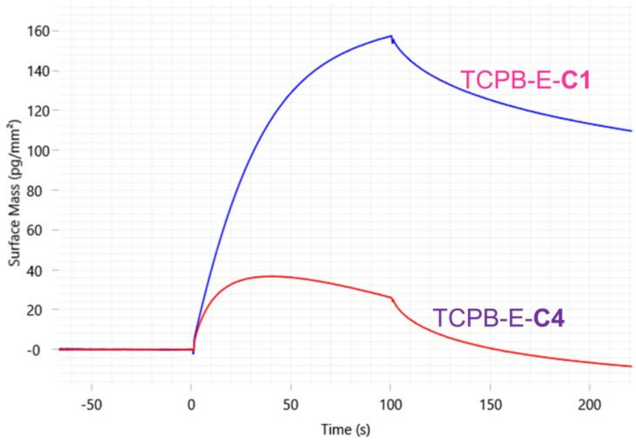

Figure S4.10: Grating-coupled interferometry (GCI) analysis of TCPB-E-**C1** and **-C4** isomers binding to D-TNFR-1 CRD2. A single cycle of association and dissociation were passed over the sensor surface, with 50 μM of each peptide conformer. Sensograms shown are blank subtracted (target flow cell - flow cell with no target). The highest sensor response with was observed for TCPB-E-**C1**, with little binding observed for TCPB-E-**C4**. The TCPB-E-**C1** conformer was thus identified as the active form of the TCPB peptide.

S5. Binding of the D-TCPB peptide to sTNFR-1

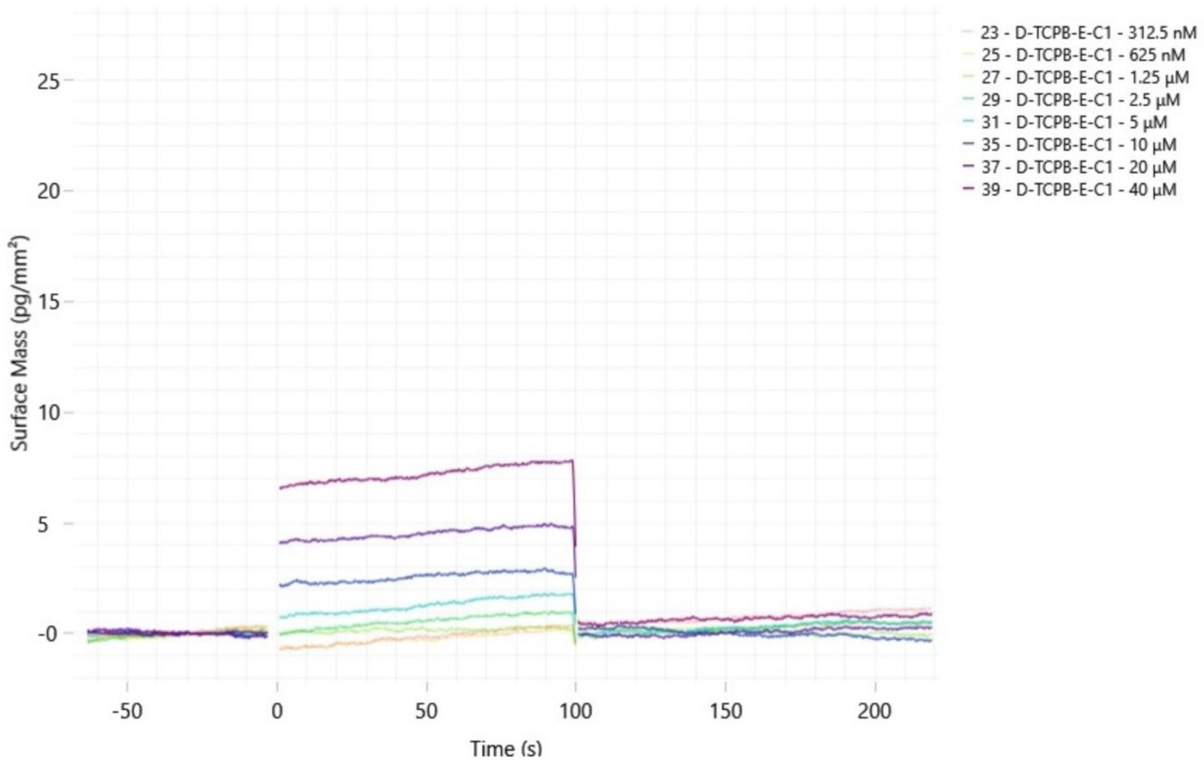

Figure S5.1: Grating-coupled interferometry (GCI) analysis of D-TCPB binding to sTNFR-1, showing weak binding affinity. Multicycle kinetics sensograms are shown at D-TCPB concentrations from 40-0.3 μM. Sensograms shown are blank subtracted (target flow cell - flow cell with no target).

S6. Experimental procedures

Materials and instruments

Unless otherwise stated, chemicals and solvents were purchased from commercial suppliers (Sigma Aldrich, Fluorochem, Acros Organics, Alfa Aesar, Cambridge Bioreagents and Fisher Scientific) and used without further purification. HPLC grade (>99.8%) dimethylformamide was used for peptide synthesis. Rink amide ProTide resin was purchased from CEM and 2-Cl-trt Fmoc-hydrazine resin was prepared as previously described.[3] LCMS data was obtained using an Agilent Infinity 1260 II HPLC system fitted with an on-line Agilent 6120 quadruple ESI-MS. Preparative HPLC was carried out using a Shimadzu Nexera preparative HPLC system. All HPLC systems used UV analyte detection at 210 nm and 280 nm. Automated SPPS was performed using a Liberty Blue microwave peptide synthesiser (CEM corp.). Peptide lyophilization was carried out by flash freezing the sample in liquid N2 and drying on a Christ Alpha 2-4 LDplus freeze-dryer. The pH measurements were conducted using a Mettler Toledo FiveEasy Plus pH meter fitted with an FP20-Micro glass electrode. For measurements in 6 M Gdn·HCl, the measured pH value was assumed to be 0.8 units lower than the actual value.[4] Circular dichroism (CD) spectra were collected using an Applied Photophysics Chirascan CD spectrometer. Protein crystallization screening was conducted using a Douglas Instruments Oryx 4 crystallization robot. Grating coupled interferometry experiments were conducted using a Creoptix WAVEsystem.

Automated SPPS of ligation fragments

Automated SPPS was conducted at a 50 μmol scale using modified CEM CarboMax coupling cycles.[5] Fmoc-amino acid stock solutions, oxyma and DIC were used at 0.2 M concentration. Reactions were stirred by N_2 bubbling for 2 seconds on, 3 seconds off.

Fmoc deprotection: Piperidine in DMF (3mL, 20% v/v) was delivered to the reaction vessel. Microwave heating proceeded as follows: 0 W 20±5 °C for 5 s, 100 W 78±2 °C for 20s, 60 W 88±2 °C for 10s, 20 W 90±1 °C for 60s. The resin was then washed with DMF (4 x 2 mL). For residues 88-93 of the N-terminal cysteine peptides, the deprotection was conducted at room temperature by delivery of piperidine in DMF (3mL, 20% v/v) for 3 minutes. The resin was drained and the deprotection repeated for 7 minutes. The resin was then washed with DMF (4 x 2 mL).
Coupling cycle: Fmoc-amino acid (0.5 mL, 2 equiv.), Oxyma (0.5 mL, 2 equiv.) and DIC (1 mL, 4 equiv.) were delivered to the reaction vessel (Final concentrations: Amino acid 50 mM, Oxyma 50 mM,

DIC 100 mM). Microwave heating proceeded as follows: 15 W 75±2 °C for 15 s, 30 W 90±1 °C for **X** s. The resin was then washed once with DMF (2 mL).

For single couplings, reactions proceeded for a total coupling time of 4 mins (**X** = **225** s). For double couplings (Arg, Cys and Val), the coupling was repeated. Due to high cost of diastereomeric D-Ile and its slower coupling rate (β-branched), a single 8 min coupling (**X** = **465** s) was implemented.

Peptide hydrazide preparation

Peptides were assembled by either manual or automated SPPS onto a 2-chlorotityl Fmoc-hydrazine resin[3] and subject to peptide cleavage. Because during automated SPPS, the mildly acidic oxyma (pK_a 4.60) can cause premature release of the peptide from a 2-Cl-(Trt) resin at 90 °C, DIPEA (20 μM) was added to the oxyma solution to minimize premature cleavage and increase yields of peptide hydrazide.[5]

C-terminal biotinylated peptide linker for TNRCD2

Rink amide ProTide resin (0.1 mmol, 0.19 mmol/g, CEM) was swollen in 50%/50% v/v DMF/DCM for 10 mins in an SPE column and drained. The Fmoc-protecting group was removed by addition of 20% piperidine in DMF (3 mL) to the resin for 2 x 5 mins. The resin was washed five times with DMF (3 mL). 2 equiv. of Fmoc-Lys(Mtt)-OH (200 mM), 1.95 equiv. of HBTU (195 mM), 2 equiv. of HOBt (200 mM) and 4 equiv. of DIPEA (400 mM) was dissolved in DMF and mixed for 0.5 min. The coupling mixture was transferred to the resin and allowed coupling to proceed for 30 mins at room temperature. The remainder of the flexible linker containing Gly-D-Ser-Gly-D-Ser-Gly was assembled using manual SPPS procedure (Section 5.3.1), with the N-terminal Fmoc group left in place. The lysine side chain protecting group, 4-methyltrityl (Mtt) was removed using 1% TFA in DCM through 14 flow washes (3 mL each), monitored qualitatively by the intense yellow colour of the Mtt-OH group. The resin was washed three times with DCM (3 mL) and three times with DMF (3mL). Biotin-N-hydroxysuccinimde ester (0.2 mmol) was dissolved in DMF (10 mL) and added to the resin along with DIPEA (0.4 mmol). The resin slurry was heated by microwave irradiation in the Liberty Blue peptide synthesizer. Microwave heating proceeded as follows: 15 W 75±2 °C for 15 s, 30 W 90±1 °C for 225 s. The resin was then washed once with DMF (2 mL) and the biotinylation reaction was repeated. The remainder of the TNFR-1 CRD2 segment (Cys88-Asn116) was assembled onto the linker using automated SPPS.

Peptide cleavage

The resin was washed with DMF (3×3 mL), DCM (3×3 mL) and Et_2O (3×3 mL). Cleavage cocktail was added to the resin and allowed to stir for 120 mins at room temperature.

Two cleavage cocktail variations were used:

Cleavage reagent B containing 8.75 mL TFA, 0.25 mL triisopropylsilane, 0.5 g phenol and 0.5 mL water was used for TCPB-E peptides following solid-phase oxidation.

Cleavage reagent K containing 8.25 mL TFA, 0.25 mL EDT, 0.5 mL H_2O, 0.5 mL thioanisole and 0.5 g phenol was used for all other peptides.

The cleavage mixture was drained from the SPE column into a 50 mL centrifuge tube and the mixture was concentrated under a stream of N_2 to <3 mL. The peptide was precipitated using ice cold Et_2O and collected by centrifugation at 3500 RCF. The crude peptide was triturated twice with Et_2O, dissolved in 1% acetic acid (20 mL) and lyophilized. Crude peptides were analysed by LCMS and purified using preparative HPLC.

Peptide LCMS analysis

Peptide samples were prepared at 0.1 mg/mL using 0.1% TFA in water and passed through a 0.22 µM nylon filter. Unless otherwise stated, samples (10 µL) were eluted with reversed mobile phase A (water + 0.1% formic acid) and B (acetonitrile + 0.1% formic acid) at 0.3 mL/min over a RP-C18 column (ACE, 2.1 mm x 100 mm, 110 Å, 3 µm) at 40 °C. A 5-70% gradient of A/B was applied over 30 minutes and analyte was detected using a UV detector at 210 nm and 280 nm, and positive electrospray ionisation mass spectrometry (ESI+ MS). ESI+ mass spectra are reported as the integrated spectra for the duration of the major peak in each UV210-nm chromatogram.

Peptide preparative HPLC

Samples were passed through a 0.22 µM nylon filter. 3-10 mL of sample was eluted with reversed mobile phase A (water + 0.1% TFA) and B (acetonitrile + 0.1% TFA) at 18 mL/min over a RP-C18 column (Shimpack GIST, 20 mm x 150 mm, 100 Å, 5µm) at room temperature. A 20-60% gradient of A/B was applied over 40 minutes and analyte was detected using a photodiode array detector at 210 nm and 280 nm. Sample fractions were collected using an automated fraction collector, their identities were confirmed by LCMS and the fractions containing the target peptide were combined and lyophilized

S7. Peptide and protein LCMS data

L-TNFR-1 CRD2 (Ser^{72}-Ser^{87}) peptide hydrazide....24

D- TNFR-1 CRD2 (Ser^{72}-Lys^{87}) peptide hydrazide....25

L- TNFR-1 CRD2 (Cys^{88}-Asn^{116}) N-cysteine peptide....26

D- TNFR-1 CRD2 (Cys^{88}-Asn^{116}) N-cysteine peptide....27

D- TNFR-1 CRD2 (Cys^{88}-Asn^{116}-Gly^{117}-Gly^{121}-Lys(biotin)^{122}) N-cysteine peptide....28

L- TNFR-1 CRD2 (Ser^{72}-Asn^{116}) reduced (not isolated)....29

L- TNFR-1 CRD2 (Ser^{72}-Asn^{116})....30

D- TNFR-1 CRD2 (Ser^{72}-Asn^{116})....31

D- TNFR-1 CRD2 (Ser^{72}-Asn^{116}-Gly^{117}-Gly^{121}-Lys(biotin)^{122})....32

L-TCPB-E-C1....33

L-TCPB-E-C4....34

L-TCPB-E-C11....35

L-TCPB-E-G11....37

L-TNFR-1 CRD2 (Ser^{72}-Ser^{87}) peptide hydrazide

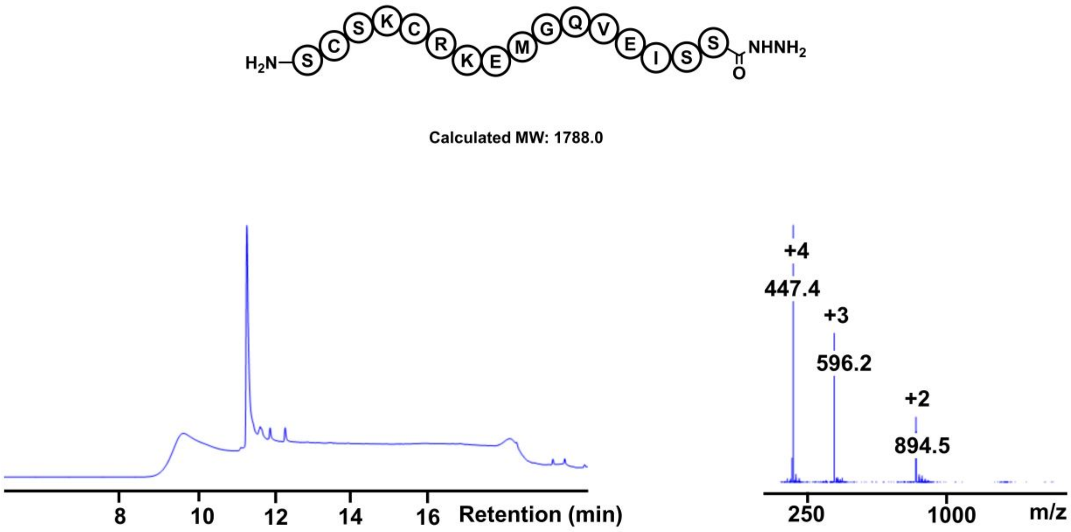

Figure S7.1: LC trace at UV210nm (left) of isolated L-TNFR-1 CRD2 (Ser^{72}-Ser^{87}) peptide hydrazide using a 0-60% gradient of A/B over 30 minutes. Right - (ESI-MS (m/z): calculated 895.0 $[M+2H]^{2+}$, 597.0 $[M+3H]^{3+}$, 448.0 $[M+4H]^{4+}$, 894.5 $[M+2H]^{2+}$, 596.2 $[M+3H]^{3+}$, 447.4 $[M+4H]^{4+}$).

D- TNFR-1 CRD2 (Ser^{72}-Lys^{87}) peptide hydrazide

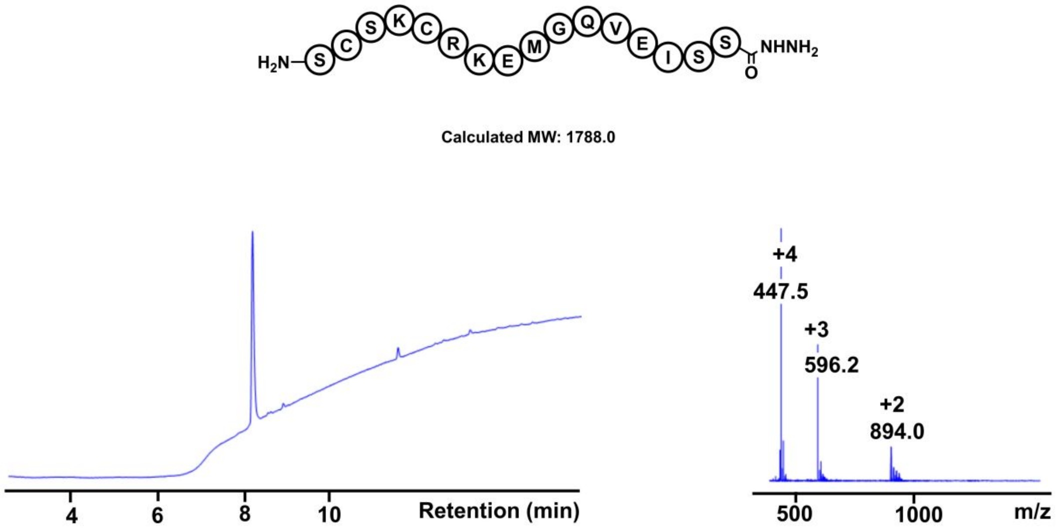

Figure S7.2: LC trace at UV210nm (left) of isolated L- TNFR-1 CRD2 (Ser^{72}-Ser^{87}) peptide hydrazide using a 5-70% gradient of A/B over 30 minutes on a RP-C18 column (Zorbax SB, 2.1 mm x 100 mm, 300 Å, 3.5 µm). Right - (ESI-MS (m/z): calculated 895.0 $[M+2H]^{2+}$, 597.0 $[M+3H]^{3+}$, 448.0 $[M+4H]^{4+}$, 894.5 $[M+2H]^{2+}$, 596.2 $[M+3H]^{3+}$, 447.4 $[M+4H]^{4+}$).

L- TNFR-1 CRD2 (Cys[88]-Asn[116]) N-cysteine peptide

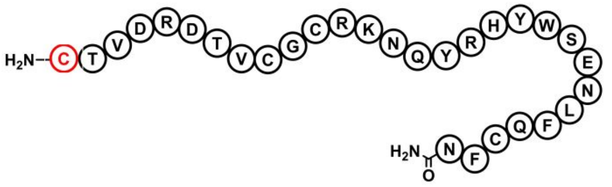

Calculated MW: 3586.0

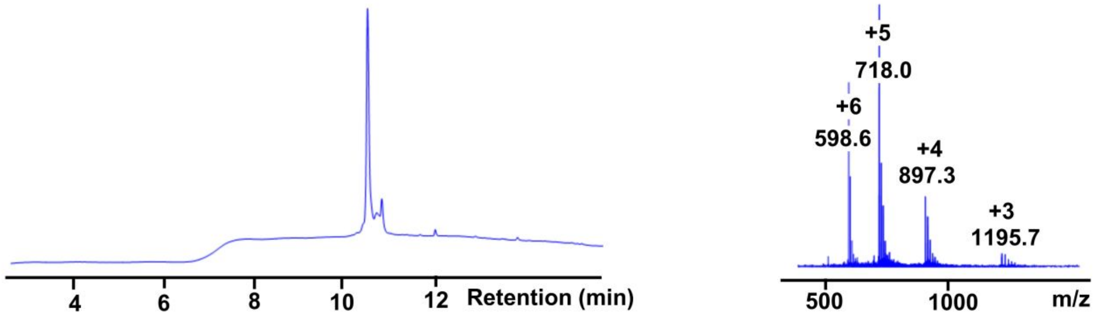

Figure S7.3: LC trace at UV210nm (left) of isolated L- TNFR-1 CRD2 (Cys[88]-Asn[116]) N-cysteine peptide using a 5-70% gradient of A/B over 20 minutes on a RP-C18 column (Zorbax SB, 2.1 mm x 100 mm, 300 Å, 3.5 µm). Right - (ESI-MS (m/z): calculated 1196.3 $[M+3H]^{3+}$, 897.5 $[M+4H]^{4+}$, 718.2 $[M+5H]^{5+}$, 598.7 $[M+6H]^{+6}$, observed 1195.7 $[M+3H]^{3+}$, 897.3 $[M+4H]^{4+}$, 718.0 $[M+5H]^{5+}$, 598.6 $[M+6H]^{+6}$).

D- TNFR-1 CRD2 (Cys^{88}-Asn^{116}) N-cysteine peptide

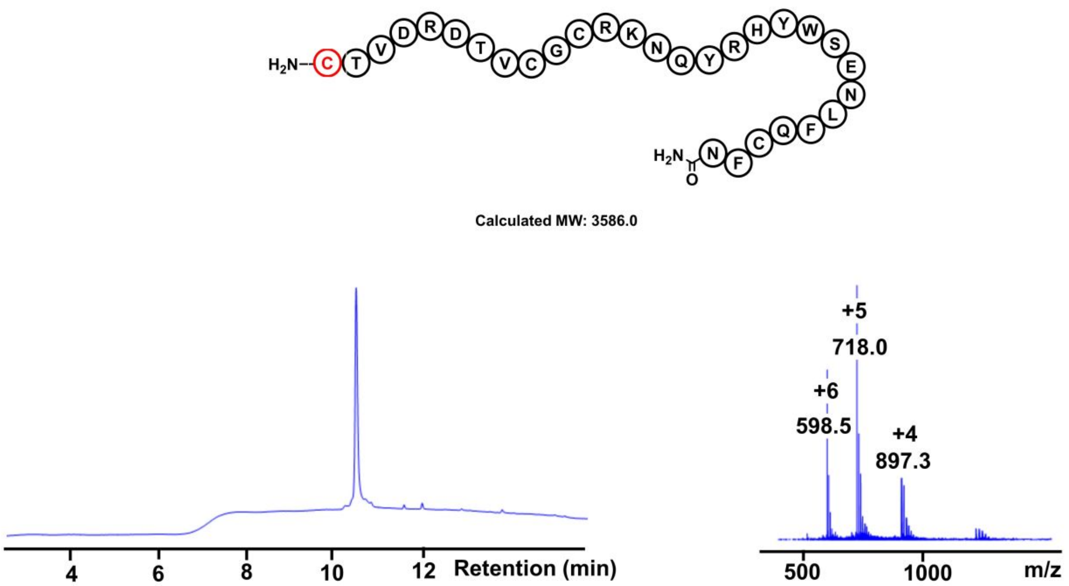

Figure S7.4: LC trace at UV210nm (left) of isolated D- TNFR-1 CRD2 (Cys^{88}-Asn^{116}) N-cysteine peptide using a 5-70% gradient of A/B over 20 minutes on a RP-C18 column (Zorbax SB, 2.1 mm x 100 mm, 300 Å, 3.5 μm). Right - (ESI-MS (m/z): calculated 897.5 $[M+4H]^{4+}$, 718.2 $[M+5H]^{5+}$, 598.7 $[M+6H]^{+6}$, observed 897.3 $[M+4H]^{4+}$, 718.0 $[M+5H]^{5+}$, 598.5 $[M+6H]^{+6}$).

D- TNFR-1 CRD2 (Cys^{88}-Asn^{116}-Gly^{117}-Gly^{121}-Lys(biotin)^{122}) N-cysteine peptide

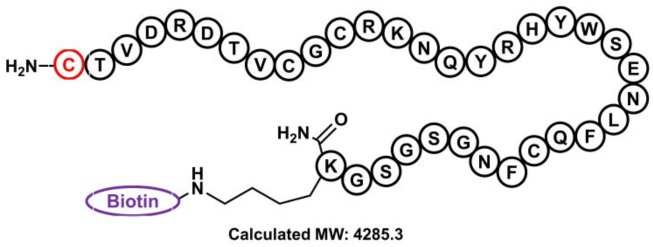

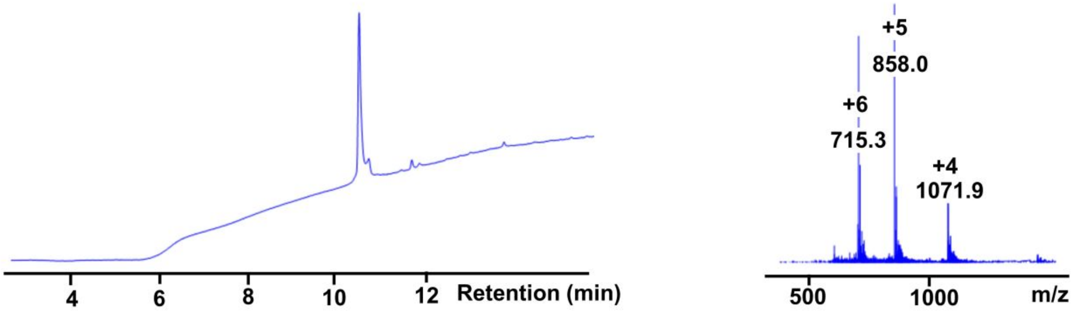

Figure S7.5: LC trace at UV210nm (left) of isolated D- TNFR-1 CRD2 (Cys^{88}-Asn^{116}-Gly^{117}-Gly^{121}-Lys(biotin)^{122}) N-cysteine peptide using a 5-70% gradient of A/B over 30 minutes on a RP-C18 column (Zorbax SB, 2.1 mm x 100 mm, 300 Å, 3.5 µm). Right - (ESI-MS (m/z): calculated 1072.3 $[M+4H]^{4+}$, 858.1 $[M+5H]^{5+}$, 715.2 $[M+6H]^{+6}$, observed 1071.9 $[M+4H]^{4+}$, 858.0 $[M+5H]^{5+}$, 715.3 $[M+6H]^{+6}$).

L- TNFR-1 CRD2 (Ser[72]-Asn[116]) reduced (not isolated)

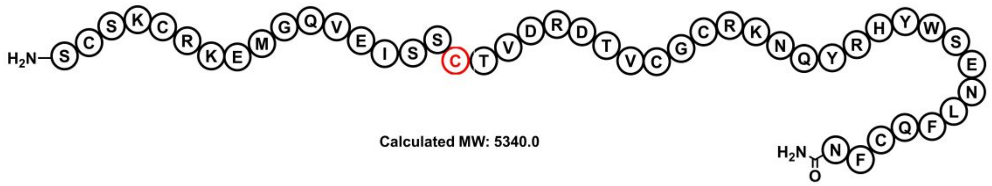

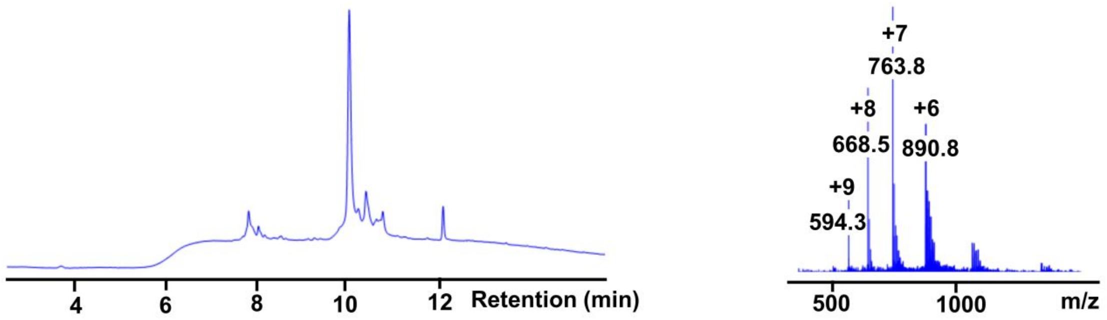

Figure S7.6: LC trace at UV210nm (left) of reduced L- TNFR-1 CRD2 (Ser[72]-Asn[116]) following native chemical ligation (without isolation). Right - (ESI-MS (m/z): calculated 897.5 $[M+4H]^{4+}$, 718.2 $[M+5H]^{5+}$, 598.7 $[M+6H]^{+6}$, observed 897.3 $[M+4H]^{4+}$, 718.0 $[M+5H]^{5+}$, 598.5 $[M+6H]^{+6}$).

L- TNFR-1 CRD2 (Ser^{72}-Asn^{116})

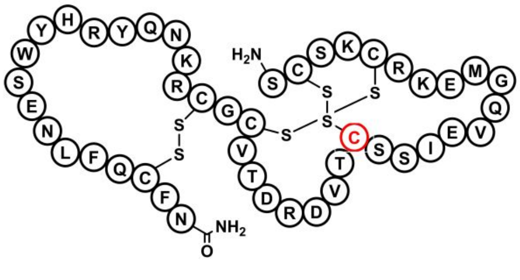

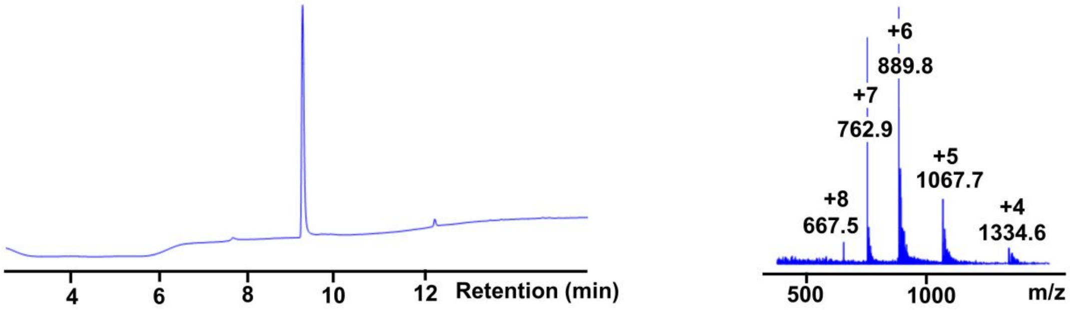

Figure S7.7: LC trace at UV210nm (left) of isolated L- TNFR-1 CRD2 (Ser^{72}-Asn^{116}). Right - (ESI-MS (m/z): calculated 1334.5 $[M+4H]^{4+}$, 1067.8 $[M+5H]^{5+}$, 890.0 $[M+6H]^{+6}$, 763.0 $[M+7H]^{+7}$, 667.7 $[M+8H]^{+8}$, observed 1334.6 $[M+4H]^{4+}$, 1067.7 $[M+5H]^{5+}$, 889.8 $[M+6H]^{+6}$, 762.9 $[M+7H]^{+7}$, 667.5 $[M+8H]^{+8}$).

D- TNFR-1 CRD2 (Ser^{72}-Asn^{116})

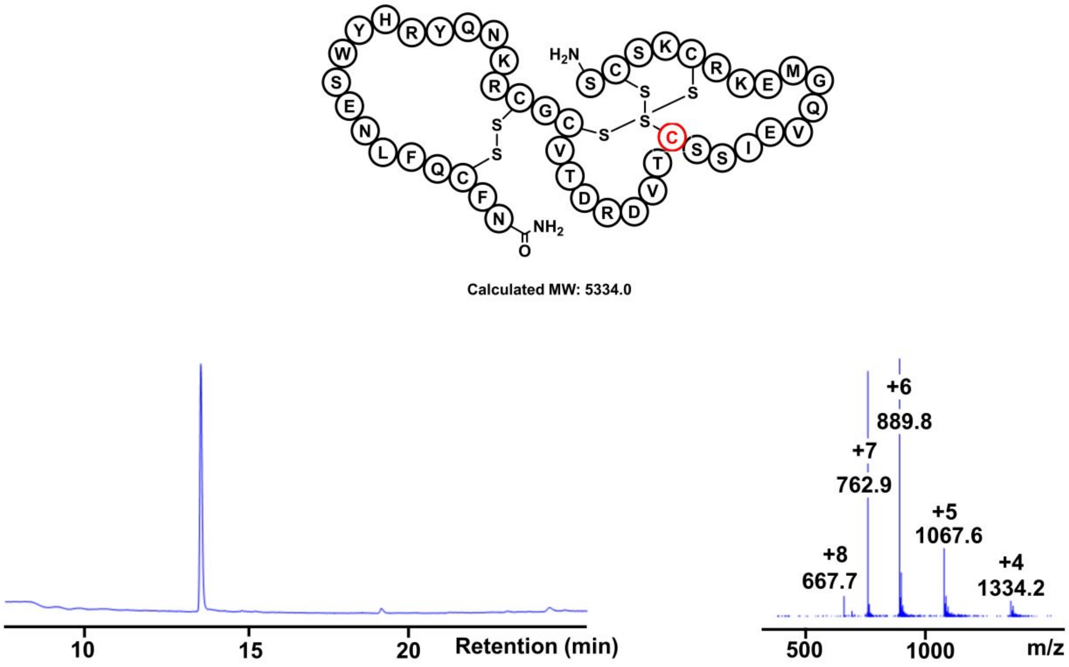

Figure S7.8: LC trace at UV210nm (left) of isolated D- TNFR-1 CRD2 (Ser^{72}-Asn^{116}) using a 5-70% gradient of A/B over 30 minutes on a RP-C4 column (ACE, 4.6 mm x 250 mm, 300 Å, 5 µm). Right - (ESI-MS (m/z): calculated 1334.5 $[M+4H]^{4+}$, 1067.8 $[M+5H]^{5+}$, 890.0 $[M+6H]^{+6}$, 763.0 $[M+7H]^{+7}$, 667.7 $[M+8H]^{+8}$, observed 1334.2 $[M+4H]^{4+}$, 1067.6 $[M+5H]^{5+}$, 889.8 $[M+6H]^{+6}$, 762.9 $[M+7H]^{+7}$, 667.7 $[M+8H]^{+8}$).

D- TNFR-1 CRD2 (Ser^{72}-Asn^{116}-Gly^{117}-Gly^{121}-Lys(biotin)^{122})

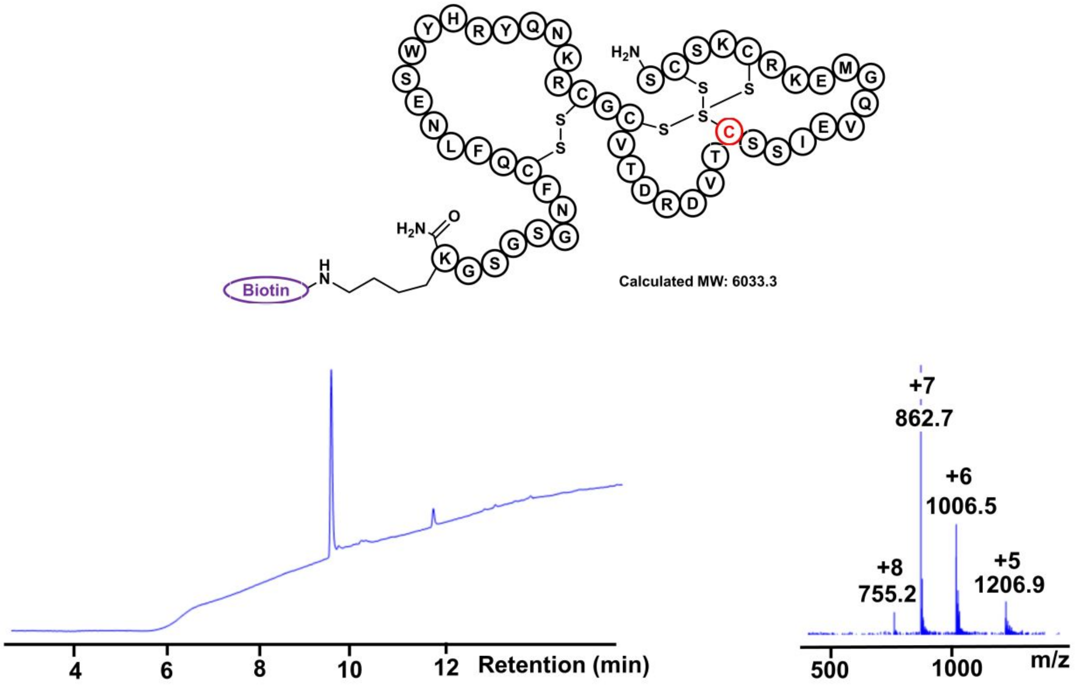

Figure S7.9: LC trace at UV210nm (left) of isolated D- TNFR-1 CRD2 (Ser^{72}-Asn^{116}-Gly^{117}-Gly^{121}-Lys(biotin)^{122}) using a 5-70% gradient of A/B over 30 minutes on a RP-C18 column (Zorbax SB, 2.1 mm x 100 mm, 300 Å, 3.5 µm). Right - (ESI-MS (m/z): calculated 1207.7 $[M+5H]^{5+}$, 1006.6 $[M+6H]^{+6}$, 862.9 $[M+7H]^{+7}$, 755.2 $[M+8H]^{+8}$, observed 1207.9 $[M+5H]^{5+}$, 1006.5 $[M+6H]^{+6}$, 862.7 $[M+7H]^{+7}$, 755.2 $[M+8H]^{+8}$).

L-TCPB-E-C1

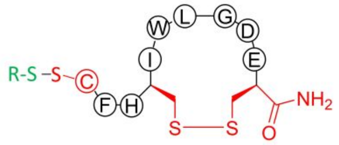

R-S = Cysteine TCPB-E-**C1**

Calculated MW: 1442.7

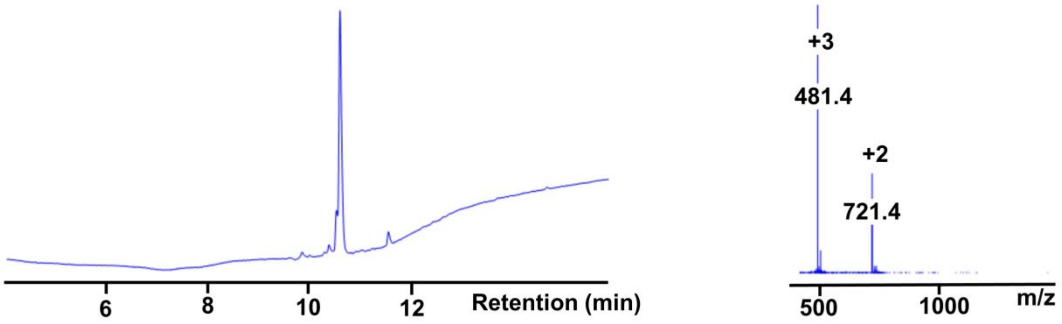

Figure S7.10: LC trace at UV210nm (left) of L-TCPB-E-C1 (isolated from solution-phase oxidation) using a 5-70% gradient of A/B over 20 minutes on a RP-C18 column (Zorbax SB, 2.1 mm x 100 mm, 300 Å, 3.5 µm). Right - (ESI-MS (m/z): calculated 722.4 $[M+2H]^{2+}$, 481.9 $[M+3H]^{+3}$, observed 721.4 $[M+2H]^{2+}$, 481.4 $[M+3H]^{+3}$).

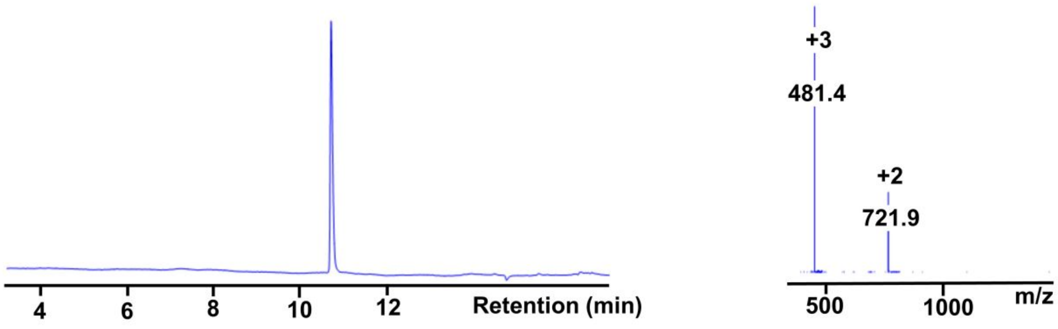

Figure S7.11: LC trace at UV210nm (left) of isolated L-TCPB-E-C1 (prepared by solid-phase oxidation, used for kinetic characterizations) using a 5-70% gradient of A/B over 20 minutes on a RP-C18 column (Zorbax SB, 2.1 mm x 100 mm, 300 Å, 3.5 µm). Right - (ESI-MS (m/z): calculated 722.4 $[M+2H]^{2+}$, 481.9 $[M+3H]^{+3}$, observed 721.9 $[M+2H]^{2+}$, 481.4 $[M+3H]^{+3}$).

L-TCPB-E-C4

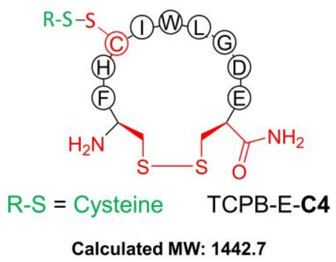

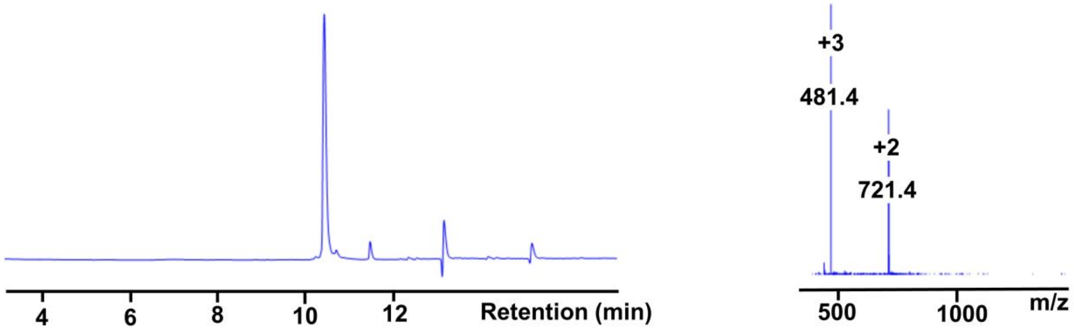

Figure S7.12: LC trace at UV210nm (left) of isolated L-TCPB-E-C4 (prepared by solid-phase oxidation) using a 5-70% gradient of A/B over 20 minutes on a RP-C18 column (Zorbax SB, 2.1 mm x 100 mm, 300 Å, 3.5 µm). Right - (ESI-MS (m/z): calculated 722.4 $[M+2H]^{2+}$, 481.9 $[M+3H]^{+3}$, observed 721.4 $[M+2H]^{2+}$, 481.4 $[M+3H]^{+3}$).

L-TCPB-E-C11

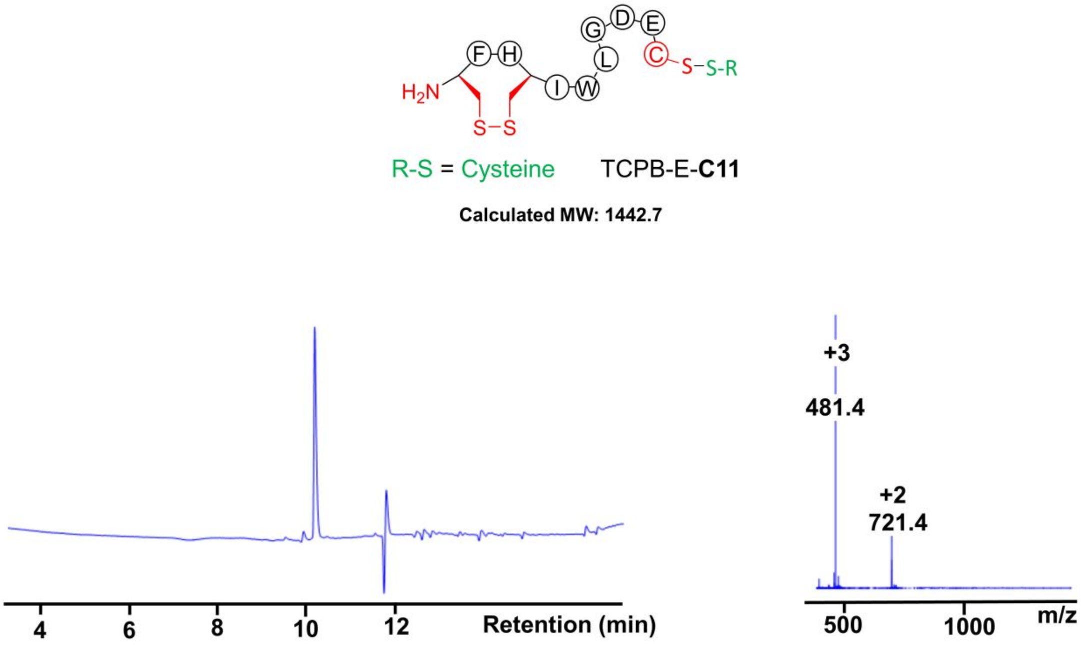

Figure S7.13: LC trace at UV210nm (left) of L-TCPB-E-C11 (isolated from solution-phase oxidation) using a 5-70% gradient of A/B over 20 minutes on a RP-C18 column (Zorbax SB, 2.1 mm x 100 mm, 300 Å, 3.5 µm). Right - (ESI-MS (m/z): calculated 722.4 $[M+2H]^{2+}$, 481.9 $[M+3H]^{+3}$, observed 721.4 $[M+2H]^{2+}$, 481.4 $[M+3H]^{+3}$).

L-TCPB-E-G1

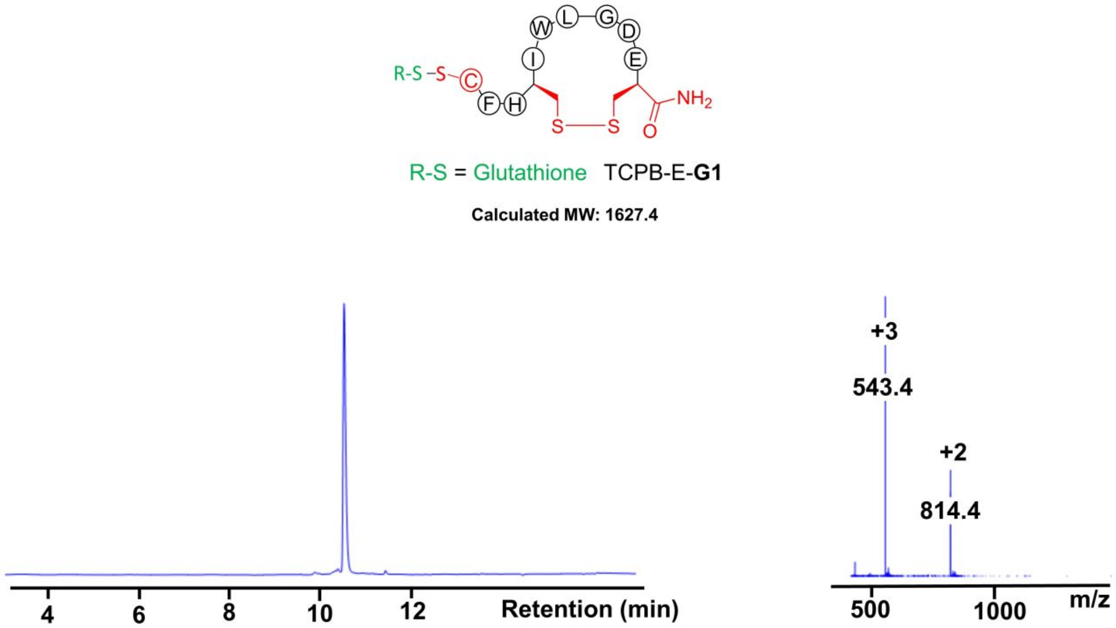

Figure S7.14: LC trace at UV210nm (left) of L-TCPB-E-G1 (isolated from solution-phase oxidation) using a 5-70% gradient of A/B over 20 minutes on a RP-C18 column (Zorbax SB, 2.1 mm x 100 mm, 300 Å, 3.5 μm). Right - (ESI-MS (m/z): calculated 814.7 [M+2H]$^{2+}$, 543.5 [M+3H]$^{+3}$, observed 814.4 [M+2H]$^{2+}$, 543.4 [M+3H]$^{+3}$).

L-TCPB-E-G11

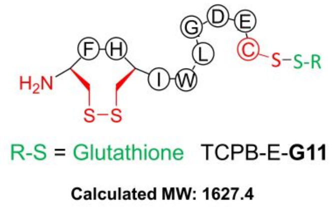

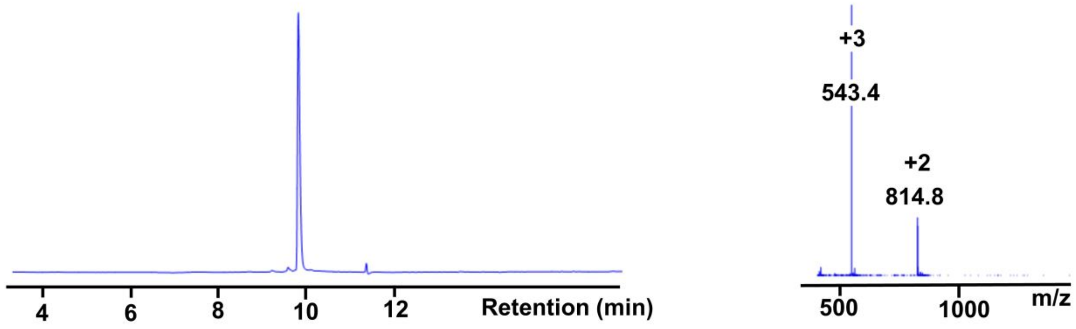

Figure S7.15: LC trace at UV210nm (left) of L-TCPB-E-G11 (isolated from solution-phase oxidation) using a 5-70% gradient of A/B over 20 minutes on a RP-C18 column (Zorbax SB, 2.1 mm x 100 mm, 300 Å, 3.5 μm). Right - (ESI-MS (m/z): calculated 814.7 $[M+2H]^{2+}$, 543.5 $[M+3H]^{+3}$, observed 814.8 $[M+2H]^{2+}$, 543.4 $[M+3H]^{+3}$).

References

(1) Anthis, N. J.; Clore, G. M. Sequence-specific determination of protein and peptide concentrations by absorbance at 205 nm. *Protein Sci* **2013**, *22* (6), 851-858. DOI: 10.1002/pro.2253 From NLM.

(2) Kamber, B.; Hartmann, A.; Eisler, K.; Riniker, B.; Rink, H.; Sieber, P.; Rittel, W. The Synthesis of Cystine Peptides by Iodine Oxidation of S-Trityl-cysteine and S-Acetamidomethyl-cysteine Peptides. *Helvetica Chimica Acta* **1980**, *63* (4), 899-915. DOI: https://doi.org/10.1002/hlca.19800630418.

(3) Huang, Y.-C.; Chen, C.-C.; Li, S.-J.; Gao, S.; Shi, J.; Li, Y.-M. Facile synthesis of C-terminal peptide hydrazide and thioester of NY-ESO-1 (A39-A68) from an Fmoc-hydrazine 2-chlorotrityl chloride resin. *Tetrahedron* **2014**, *70* (18), 2951-2955. DOI: https://doi.org/10.1016/j.tet.2014.03.022.

(4) Garcia-Mira, M. M.; Sanchez-Ruiz, J. M. pH corrections and protein ionization in water/guanidinium chloride. *Biophys J* **2001**, *81* (6), 3489-3502. DOI: 10.1016/s0006-3495(01)75980-2 From NLM.

(5) Collins, J. M.; Porter, K. A.; Singh, S. K.; Vanier, G. S. High-Efficiency Solid Phase Peptide Synthesis (HE-SPPS). *Organic Letters* **2014**, *16* (3), 940-943. DOI: 10.1021/ol4036825.
